# Loss of regulation of protein synthesis and turnover underpins an attenuated stress response in senescent human mesenchymal stem cells

**DOI:** 10.1073/pnas.2210745120

**Published:** 2023-03-29

**Authors:** Jack Llewellyn, Venkatesh Mallikarjun, Ellen Appleton, Maria Osipova, Hamish T. J. Gilbert, Stephen M. Richardson, Simon J. Hubbard, Joe Swift

**Affiliations:** ^a^Wellcome Trust Centre for Cell-Matrix Research, Division of Cell Matrix Biology and Regenerative Medicine, School of Biological Sciences, Faculty of Biology, Medicine and Health, Manchester Academic Health Science Centre, University of Manchester, Manchester M13 9PT, United Kingdom; ^b^Division of Cell Matrix Biology and Regenerative Medicine, School of Biological Sciences, Faculty of Biology, Medicine and Health, Manchester Academic Health Science Centre, University of Manchester, Manchester M13 9PL, United Kingdom; ^c^Division of Evolution and Genomic Sciences, School of Biological Sciences, Faculty of Biology, Medicine and Health, Manchester Academic Health Science Centre, University of Manchester, Manchester M13 9PL, United Kingdom

**Keywords:** proteostasis network, mesenchymal stem cells, molecular chaperones, proteomics, senescence

## Abstract

Cells respond to stress by synthesizing chaperone proteins that seek to correct protein misfolding and maintain function. However, abrogation of protein homeostasis is a hallmark of aging, leading to loss of function and the formation of proteotoxic aggregates characteristic of pathology. Consequently, discovering the underlying molecular causes of this deterioration in proteostasis is key to designing effective interventions to disease or to maintaining cell health in regenerative medicine strategies. Here, we examined primary human mesenchymal stem cells, cultured to a point of replicative senescence and subjected to heat shock, as an in vitro model of the aging stress response. Multi -omics analysis showed how homeostasis components were reduced in senescent cells, caused by dysregulation of a functional network of chaperones, thereby limiting proteostatic competence. Time-resolved analysis of the primary response factors, including those regulating heat shock protein 70 kDa (HSPA1A), revealed that regulatory control is essentially translational. Senescent cells have a reduced capacity for chaperone protein translation and misfolded protein (MFP) turnover, driven by downregulation of ribosomal proteins and loss of the E3 ubiquitin ligase CHIP (C-terminus of HSP70 interacting protein) which marks MFPs for degradation. This limits the cell’s stress response and subsequent recovery. A kinetic model recapitulated these reduced capacities and predicted an accumulation of MFP, a hypothesis supported by evidence of systematic changes to the proteomic fold state. These results thus establish a specific loss of regulatory capacity at the protein, rather than transcript, level and uncover underlying systematic links between aging and loss of protein homeostasis.

## Significance

Cellular senescence and the loss of protein homeostasis are hallmarks of aging, with the former linked to lost efficacy in regenerative medicine and the latter a precursor for age-associated protein folding diseases. Here, using a combination of targeted, -omic, and mathematical approaches, we show how senescent human mesenchymal stem cells are less equipped to resolve the high levels of protein misfolding typical of aged tissue and the post-transplant environment. We report that a lack of translational capacity and a downregulation of protein turnover machinery led to loss of the speed, magnitude, and efficacy of the cellular stress response in senescent cells. Supported by mathematical modeling, these findings challenge a long-standing paradigm of transcriptionally driven regulation of the cellular stress response.

Proteins are molecular machines required for cell and tissue function, providing structure and performing vital transport, signaling, and enzymatic roles. The proteome must be actively regulated to match functional demands and address challenges—a state of proteostasis—thus ensuring that proteins are correctly folded, present in the right locations at the appropriate concentrations, and with any necessary post-translational modifications (1). Due to its importance, proteostasis is safeguarded by a coordinated “proteostasis network” (PN) that executes several functions: Molecular chaperones assist in the folding of newly synthesized proteins and resolve misfolding events as part of the cellular stress response, while protein degradation machinery allows misfolded or surplus proteins to be removed or recycled. Dysregulation of the PN is a recognized consequence of aging (2, 3). Aged cells have decreased chaperone and proteasomal activity and consequently accumulate oxidatively damaged and misfolded proteins (MFPs) (4). The detrimental effects of protein misfolding are two-fold: First, the loss of a protein’s structure leads to the loss of its function; and second, the MFPs can form aggregates that are toxic to cells. Protein aggregation is characteristic of diseases such as Alzheimer’s, Parkinson’s, and Huntington’s—all disorders where age is considered a major risk factor (5). Progress in the development of drugs to address diseases such as Alzheimer’s has been slow (6), but a better understanding of the PN may inform new therapeutic strategies.

The PN in human cells contains of the order of two thousand component proteins (7). Within this group, ~300 chaperone and cochaperone proteins (referred to as the “chaperome”) are associated with protein folding and conformational maintenance (8). Chaperone proteins function by guiding their partially folded client proteins through a free-energy landscape to the global energetic minimum associated with the native folded state. In mechanistic terms, this process requires that chaperones shield exposed hydrophobic regions of their partially folded clients, thus preventing unwanted interactions (9). Many proteins within the chaperome are classified as heat shock proteins (HSPs) as they are expressed in response to heat as a prototypic form of stress and are grouped into families by their molecular weight (kDa). Proteins within the HSP60, HSP70, HSP90, and HSP100 families have activity dependent on adenosine triphosphate (ATP) metabolism, whereas the small HSPs are ATP-independent (10). Chaperone function is further regulated by cochaperone proteins, such as the tetratricopeptide repeat proteins that assist the HSP90 system and the HSP40 family of proteins that increase the client-specificity of HSP70 chaperones (11). The expression of many HSPs is coordinated by the heat shock response (HSR) pathway: here, an accumulation of MFP causes disassembly of complexes of HSPs, allowing them to engage with client proteins. This disassembly process releases the transcription factor heat shock factor 1 (HSF1), allowing it to translocate to the nucleus where it acts as a master regulator of HSP transcription.

More than one hundred genes related to the PN have been shown to be significantly suppressed with age in humans (8). Correspondingly, chaperone-assisted protein folding and disaggregation processes have been demonstrated to deteriorate with aging (4, 12). The protective response to heat stress was found to deteriorate with aging in *Caenorhabditis elegans*, concomitant with increased levels of protein unfolding (13). In the same model, knockdown of HSF1 was found to amplify age-associated loss of protein function, while its overexpression extended the maintenance of proteostasis and lengthened lifespan (13, 14). In post-mitotic tissues of mice (heart, spleen, renal, and cerebral cortices), basal levels of HSP70 protein were found to be decreased in aged vs. adult animals. Interestingly, however, adult levels of HSP70 were found to be maintained in naturally long-lived animals (15). A study of human-derived lymphoblasts has shown that the ability to increase levels of HSP70 gene expression in response to stress was generally decreased by aging but that the response was maintained in cells from exceptionally long-lived subjects (16). Taken together, this evidence suggests that dysregulation of PN components with aging is widely conserved, that age may affect both basal levels of HSPs and their ability to respond to stress, and that prolonged maintenance of proteostasis machinery may benefit both health and longevity. Nonetheless, a systematic survey of how the complex PN responds to stress, and how the response is affected by aging, remains lacking—particularly in human cells and tissues.

The effects of aging on human physiology vary greatly between individuals due to the underlying genetic and environmental diversity. Consequently, paired studies rather than global comparisons between groups of young and aged individuals are preferable. A lack of these longitudinal aging studies has limited our understanding of the process. Cellular senescence, such as achieved through replication of primary cells, has therefore often been used as an in vitro aging model (17). Senescence is a cellular response to irreparable DNA damage: to prevent propagation of potentially oncogenic damage the cell cycle is arrested and the cell marked for clearance. Changes to relative rates of cell damage, repair, clearance and renewal mean that aged tissues have disproportionately high numbers of senescent cells compared to young tissues (17). Senescent cells have correspondingly been identified as a “hallmark of aging” (18) and have been reported to have decreased levels of HSPs (19) and lowered proteasomal (20) and mitochondrial activity (17).

In this study, we have examined the response of primary human mesenchymal stem cells (hMSCs) to heat stress. MSCs can be derived from a broad range of tissues, including bone marrow and adipose tissue (21) and have been widely studied in the context of tissue engineering and regenerative medicine, with a range of applications currently subject to clinical trials (22). Consequently, this study has important implications to this purpose because: i) how aging and/or serial expansion in culture impacts on hMSC behavior is an important consideration when applying autologous treatment strategies to older patients—indeed, MSCs sourced from bone marrow, commonly used in clinical applications, have been shown to have greater propensity toward senescence in serial expansion than MSCs derived from adipose tissue (23); and ii) many medical applications of hMSCs will require that the cells be robust to stress (24), and inflammation, infection, and tissue injury/repair are known triggers of the HSF1-mediated stress response (25). We have used replicative senescence to model the effects of aging, comparing early passage (EP) and senescent hMSCs from matched donors to maximize the statistical power of our analysis. We have constructed a systematic, time-resolved characterization of how senescence affects the speed, magnitude, and efficacy of the stress response. Furthermore, we have applied network and computational analysis to better understand the complexity of the PN and model the response to stress. We report on the senescence-induced decline of a functional module within the chaperome, centered around the activity of heat shock protein family A (HSP70) member 1A (HSPA1A), and attribute a weakening of the responsiveness to stress to depreciation of translational and protein turnover capacity. In summary, this investigation into the nature and cause of the decline of the HSR in senescent hMSCs provides mechanistic insights into how proteostasis is lost and offers a starting point for strategies that could recover it.

## Results

### The Proteome Is Affected Broadly by the Onset of Senescence.

**Fig. 1.**
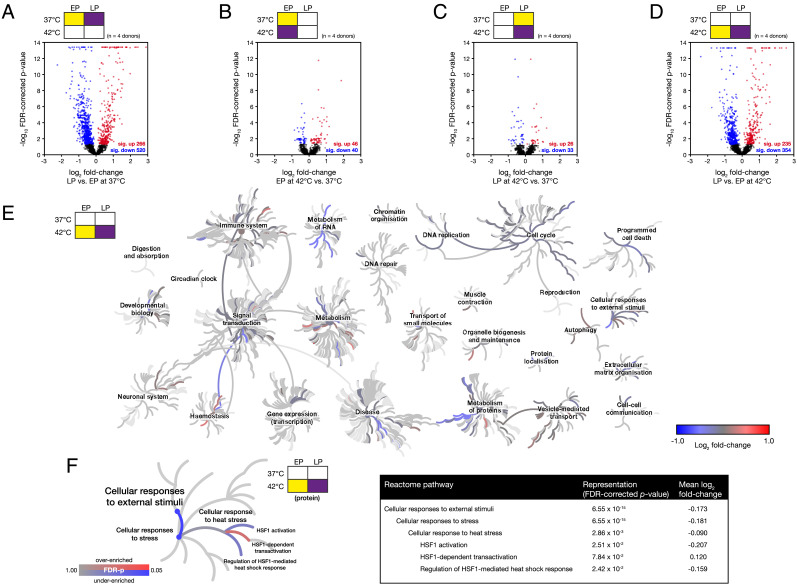
Proteomic analysis shows that senescent human mesenchymal stem cells (hMSCs) have a supressed response to thermal stress. Proteomic analysis of whole-cell lysates of donor-matched early passage (EP) and senescent late passage (LP) hMSCs with and without a 2-h heat shock treatment at 42 °C. Volcano plots show the distribution of changes in the abundance of 1,830 proteins in (*A*) LP vs. EP hMSCs without heat shock; (*B*) EP hMSCs with and without heat shock; (*C*) LP hMSCs with and without heat shock; (*D*) LP vs. EP hMSCs, both subjected to heat shock. In figure panels *A*–*D*, red and blue points satisfy *P* < 0.05. *P* values were calculated using empirical Bayes-modified *t* tests with Benjamini–Hochberg false discovery rate (FDR) correction (29); *n* = 4 primary donors. (*E*) Reactome pathway analysis of the significantly affected proteins shown in panel *D*, comparing the responses of EP and LP hMSCs subjected to heat shock (30). Significantly represented pathways (FDR-corrected *P* value < 0.05) are shown in colours corresponding to the mean log_2_ fold-change of proteins in the pathway. (*F*) Expanded view of the pathway “cellular response to heat stress” and its parent and child pathways. The six coloured pathways of interest were subjected to an FDR-corrected PANTHER statistical enrichment analysis (31).

We first sought to characterize the intracellular proteomes of proliferating, EP and senescent, late passage (LP) primary hMSCs under control conditions (culture at 37 °C). EP hMSCs were used between passages 1 and 7, while the onset of senescence in LP hMSCs occurred between passages 5 and 18. Senescence was confirmed in LP cells by positive β-galactosidase staining (*SI Appendix*, Fig. S1*A*), expression of β-galactosidase protein (*P* < 0.0001, false discovery rate (FDR)-corrected ANOVA; *SI Appendix*, Fig. S1*B*), and loss of lamin B1 protein (LMNB1; *P* < 0.0001, FDR-corrected ANOVA) and transcript (*LMNB1; P* < 0.0001, *t* test; *SI Appendix*, Fig. S1 *B* and *C*) (26). LP cells were also found to have significantly greater spread areas than EP cells (*P* ≤ 0.008, *t* tests) and generally had reduced aspect ratios, consistent with a loss of spindle-like morphology (*SI Appendix*, Fig. S1 *D* and *E*)—both characteristic of senescence (27). Although nuclear spreading has been found to correlate with cellular spreading in other contexts, such as during mechanical engagement with the substrate (28), this relationship was not reliably conserved in LP vs. EP (*SI Appendix*, Fig. S1*F*). Log_2_ fold-changes in protein level were quantified in whole-cell lysates using liquid chromatography-coupled tandem mass spectrometry (LC-MS/MS) (29), comparing donor-matched LP vs. EP hMSCs; of 1,830 proteins identified with ≥3 peptides-per-protein, levels of 286 were found to be significantly increased and 520 significantly decreased (*P* < 0.05, FDR-corrected ANOVA; Fig. 1*A*). Ontological analysis using the Reactome database (30) showed significant decrease of “cell cycle”, “DNA replication”, and “translation” pathways (FDR-corrected *P* values < 0.05), suggesting suppression of processes consistent with the “hallmarks of cellular senescence” (27). Interestingly, both “cellular response to heat stress” and “regulation of HSF1-mediated HSR” pathways were significantly diminished in LP vs. EP hMSCs, even in the absence of heat stress.

### The Response to Heat Stress Is Attenuated in Senescent Cells.

Having characterized the effects of senescence on the hMSC proteome, we examined the responses of EP and LP cells to heat stress treatment. Using LC-MS/MS to again quantify changes to 1,830 proteins detected with ≥3 peptides-per-protein, we compared hMSCs subjected to a 2-h treatment at 42 °C to matched cells under control conditions. Using EP hMSCs, we found that heat stress caused a significant increase of 46 proteins and decrease of 40 proteins (*P* < 0.05, FDR-corrected ANOVA; Fig. 1*B*). The number of significantly affected proteins was decreased in LP hMSCs subjected to the same treatment: 26 increased and 33 decreased (*P* < 0.05, FDR-corrected ANOVA; Fig. 1*C*). Examination of affected Reactome pathways showed that “cellular response to heat stress” and “regulation of HSF1-mediated HSR” were significantly increased in response to heat stress in both EP and LP hMSCs (FDR-corrected *P* values < 0.05); however, the mean fold-change to protein levels in these pathways was decreased in LP cells, indicative of a suppressed stress response. Notably, the perturbation to the proteome caused by heat shock to EP or LP hMSCs was considerably smaller than the change following the onset of senescence (comparing Fig. 1 *A*–*C*). We therefore compared the proteomic endpoints of both EP and LP hMSCs following the heat treatment, as means of contrasting their relative capacities to manage proteotoxic stress. In addition to generating a volcano plot of LP vs. EP hMSCs following 2-h heat treatment at 42 °C (Fig. 1*D*), we mapped the mean log_2_ fold-changes of proteins within significantly affected Reactome pathways onto a network capturing all cellular processes (FDR-corrected *P* values < 0.05, Fig. 1*E*). This plot again highlights broad proteomic differences between EP and LP hMSCs—attributed to processes such as metabolism of proteins, cell cycle, apoptosis, and organization of the extracellular matrix—but also suggests a failure to adequately respond to proteotoxic stress following senescence: The parent pathway “cellular responses to external stimuli” was significantly affected (FDR-corrected *P* values < 0.0001; shown expanded in Fig. 1*F*), and within that, both “cellular response to heat stress” and “regulation of HSF1-mediated HSR” were suppressed. Interestingly, while the parent pathways “metabolism” and “transport of small molecules” were not found to be significantly affected, the respective subpathways “metabolism of carbohydrates” and “iron uptake and transport” were significantly overenriched (FDR-corrected *P* values < 0.05; *SI Appendix*, Fig. S1*G*), consistent with characteristics of senescence (27, 32).

### The Human Chaperome Can Be Divided into Functional Modules Based on Protein–Protein Interactions (PPI).

**Fig. 2.**
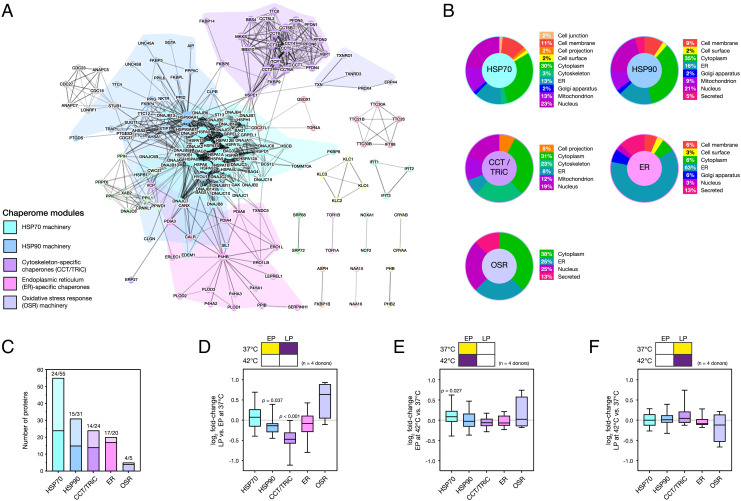
Network analysis of the chaperome shows that the heat stress response of a heat shock protein 70 kDa (HSP70) module is lost in late passage (LP) human mesenchymal stem cells (hMSCs). (*A*) The community structure of proteins identified in the human chaperone network (8). Proteins (nodes) are colored by their inclusion into modules, with the five largest modules named according to their function and listed in the key. Node size is indicative of chaperone degree (*SI Appendix*, Fig. S2*A*), while edge weight indicates the STRING interaction score between chaperones (*SI Appendix*, Fig. S2*B*) (33). (*B*) Distributions of the cellular locations of chaperones in each module, determined from UniProt annotations (40). (*C*) Plot showing the number of constitutive proteins from each chaperome module detected in the proteomics dataset presented in Fig. 1. (*D*) Fold-changes to proteins in chaperome modules in donor-matched LP vs. EP hMSCs without heat shock treatment. (*E*) Fold-changes to proteins in chaperome modules in EP hMSCs subjected to a 2-h heat shock treatment at 42 °C. The HSP70 module was significantly increased (*P* = 0.03). (*F*) Fold-changes to proteins in chaperome modules in LP hMSCs subjected to heat shock; none of the modules were significantly regulated. In figure panels *D*–*F*, box-whisker plots show medians, quartiles, and range; *P* values from ANOVA; *n* = 4 primary donors.

In order to better understand the consequences of suppressed stress response in senescent cells, we sought to characterize the roles of individual functional modules within the chaperome. The human chaperome described by Brehme et al. consists of 332 proteins; inclusion within this network of chaperone and cochaperone proteins was based on analysis of the literature, domain structures, and annotations of human and *C. elegans* genomes (8). Using the highest-confidence PPI data taken from the STRING database (33), we performed modularity analysis (34) on the Brehme et al. chaperome to subdivide it into deeply interconnected modules (*SI Appendix*, Fig. S2 *A* and *B*). The question we sought to answer was—in physical terms—whether all chaperones had the broad remit of maintaining proteostasis as a collective (i.e., low modularity) or whether chaperones could be separated into functional modules that perform more specialized tasks (i.e., high modularity). We found the human chaperone network to be highly modular, dividing into 19 communities (Fig. 2*A*). These communities did indeed seem to be dedicated to specific tasks within the maintenance of proteostasis: Nearly all members of the HSPA and DNAJ families of proteins were grouped together into one module, which we refer to as “HSP70 machinery”. Similarly, a module containing the two human HSP90 proteins and several documented HSP90 cochaperones (35) were grouped together (“HSP90 machinery”). Chaperones localized in the endoplasmic reticulum (ER) were clustered together in a module containing members of the P4HA and PLOD families of proteins (“ER-specific chaperones”); chaperonin containing t-complex/TCP1 ring complex (CCT/TRiC) proteins, linked to cell cycle progression and maintenance of microtubule and microfilament components of the cytoskeleton (36), were also grouped (“cytoskeleton-specific chaperones”). Another module consisted of the reductases TXN, TXNRD1, TXNRD3, PRDX4, and ERP44, which protect against oxidative stress (37) and has been named as such (“oxidative stress response machinery”). The ERP44–PRDX4 complex has a recognized role in oxidative protein folding (38), but has also been shown to protect ribosomes against stress-induced aggregation (39). An analysis of the localizations of chaperones in each module using UniProt annotations (40) gives further insight into these modules (Fig. 2*B*). The HSP70 machinery is the least location-specific of the chaperone modules, followed by the HSP90 machinery, suggestive of promiscuous functionality within the cell. The module containing CCT/TRiC components has an even split between the cytoplasm (31%) and cytoskeleton (23%). Interestingly the oxidative stress response module, made up of oxidoreductases, had the largest percentage of localization annotations within the nucleus (25%). These contrasting distributions of the chaperone modules throughout the cell further highlight their distinct functions. We next analyzed functional annotations within the individual interacting communities, focusing on the five modules most represented by our LC-MS/MS data.

### A Module Based around HSP70 Machinery Becomes Unresponsive to Heat Stress in Senescent Cells.

Having established how the chaperome could be subdivided into interacting functional groups, we reanalyzed our initial proteomic characterization of the HSR in EP and LP hMSCs (Fig. 1). We found that the 1,830 proteins detected with ≥3 peptides-per-protein in our proteomics dataset gave representative coverage of the five main chaperome groups identified by modularity analysis (minimum coverage of 43%, Fig. 2*C*). Analysis of the data comparing LP vs. EP hMSCs in the absence of heat stress showed significant downregulation of two functional chaperome modules in senescence: HSP90 machinery (*P* = 0.04, ANOVA) and cytoskeleton-specific proteins (*P* < 0.001, ANOVA) (Fig. 2*D*). Given the role of CCT/TRiC proteins in protection of the cytoskeleton, its substantial downregulation in senescence may be of consequence to the mechanical changes that occur to cells and tissues during aging (41). Nonetheless, when we examined the response of EP hMSCs to heat stress, we found only the module associated with HSP70 machinery to have increased (*P* = 0.03, ANOVA; Fig. 2*E*). The HSP70 machinery module contains proteins from the HSPA family that were found to have the highest number of interactions in our modularity analysis, indicating that they are the key nodes within the chaperome network (*SI Appendix*, Fig. S2*A*) (42). Heat shock cognate 71 kDa protein (HSPA8) had 64 protein-protein interactions, the greatest number in the network; HSP 70 kDa (HSPA1A) had 47 network interactions and was also the most stress-sensitive protein within the module (*SI Appendix*, Fig. S2*C*). In contrast, when senescent hMSCs were subjected to the same heat stress treatment, there was no significant response in any of the chaperome modules (Fig. 2*F*); in comparison to EP cells, fold-changes to proteins constitutive of the HSP70 machinery module were generally supressed (*SI Appendix*, Fig. S2*D*). Together, this evidence demonstrates that in EP hMSCs, upregulation of HSP70 machinery is the central feature in cellular management of the proteotoxic shock caused by heat stress but that senescent hMSCs are no longer as capable of this response. In order to further demonstrate the use of the chaperome network analysis and explore crosstalk between functional modules, we examined the effects of chaperone-specific inhibitors; discussion of these results can be found in *SI Appendix*.

### Senescence Suppresses the Expression of HSPA1A Protein following Heat Stress.

**Fig. 3.**
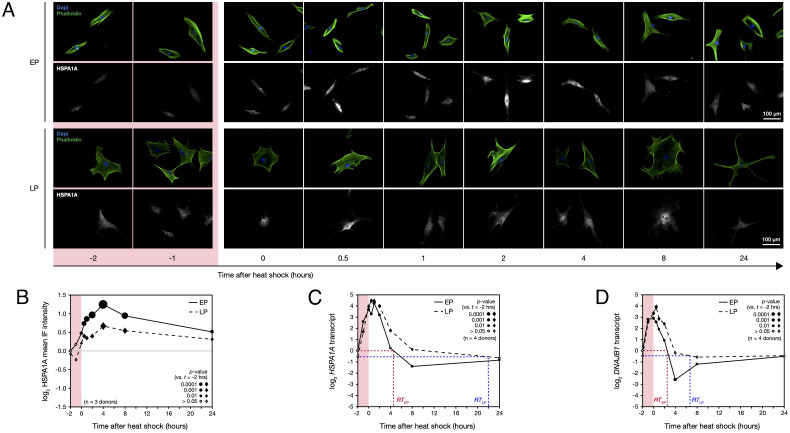
Early passage (EP) human mesenchymal stem cells (hMSCs) induce significantly more heat shock protein 70 kDa (HSPA1A) in response to heat stress than late passage (LP) cells. (*A*) Representative immunofluorescence (IF) images of heat shock protein 70 kDa (HSPA1A) in EP and LP hMSCs before, during, and for 24 h following a 2-h heat shock treatment at 42 °C. (*B*) Quantification of mean HSPA1A intensities in images shown in panel *A*. Size of points indicates significance of change in protein level vs. preheat shock, with solid points showing *P* < 0.05 (ANOVA, *n* = 3 primary donors). (*C*) Corresponding levels of heat shock protein 70 kDa transcript (*HSPA1A*) in EP and LP hMSCs before, during, and following heat stress. The recovery time (RT) required to return to prestress levels of *HSPA1A* was ~4 h in EP hMSCs, but 22 h in LP cells. (*D*) Levels of DnaJ homolog subfamily B member 1 transcript (*DNAJB1*). The RT to prestress levels was ~2 h in EP hMSCs, vs. ~6 h in LP. In panels *C* and *D*, point size indicates significance of change in transcript level vs. preheat shock, with solid points showing *P* < 0.05 (ANOVA, *n* = 4 primary donors).

In order to build a mechanistic understanding of the difference between the stress response in EP and LP hMSCs, we sought to examine key components of the heat stress response with finer temporal resolution. HSPA1A was visualized by immunofluorescence (IF) microscopy before, halfway through, and immediately following a 2-h heat treatment at 42 °C, and at timepoints over a 24-h recovery period (Fig. 3*A*). Subsequent quantification of mean HSPA1A IF intensity (analogous to protein concentration in the cell) showed that protein levels increased significantly during the heat treatment, reached a maximum 4 h into the recovery period, and then slowly began to decrease (Fig. 3*B*). This was the case in both EP and LP hMSCs, with the levels of HSPA1A prior to heating not differing significantly. However, the initial production of HSPA1A was delayed by approximately an hour in the LP response and reached a significantly lower maximum—only 63% of that found in EP cells (see Fig. 3*B* and *SI Appendix*, Fig. S3*A* for significance values, determined by ANOVA). An interesting property of HSPA1A is that it is known to be rapidly and reversibly translocated to the nucleus in cells subjected to thermal stress (43). By separately quantifying HSPA1A levels in the nucleus and cytoplasm (demarcated by DAPI and phalloidin staining, Fig. 3*A*), we found that nuclear HSPA1A levels mirrored those characterized in the rest of the cell: They reached a maximum after 4 h, and levels were lower in LP than EP hMSCs (*SI Appendix*, Fig. S3 *B* and *C*). We also examined the ratio between nuclear and cytoplasmic HSPA1A levels, finding significant differences between the behaviors of EP and LP cells, both during heat treatment and in the following 2 h (*SI Appendix*, Fig. S3 *D* and *E*). This is suggestive of changes in the regulation of HSPA1A, its transport, and/or its client protein interactions and consequently that senescence may affect how chaperone resources are directed.

### Senescence Has Minimal Impact on Transcriptional Regulation of the HSR.

We next examined whether the failure of LP hMSCs to match the magnitude of the HSPA1A stress response in EP cells was due to supressed levels of transcript. However, quantification of *HSPA1A* transcript by RT-qPCR showed the response in EP and LP hMSCs to be remarkably similar: Starting from comparable prestress levels, in both cases, transcripts rose rapidly during heat treatment and reached a similar maximum level an hour into the recovery period (Fig. 3*C*). The primary difference between the behavior of EP and LP cells was the time required to return to the prestress transcript level [recovery time (RT) *RT_EP_* vs. *RT_LP_*, indicated on Fig. 3*C*]: in EP cells, *HSPA1A* was insignificantly different from the initial level after 4 h of recovery; in LP cells, this RT was greater than 8 h (significance from ANOVA indicated on Fig. 3*C*). The time difference between peaks in *HSPA1A* transcript and levels of the encoded protein was about 3 h (comparing Fig. 3 *B* and *C*), consistent with earlier characterizations of the heat stress response in yeast (44). This delay implies that an initial regulation of HSPA1A must be posttranslational. We also quantified the stress response of *DNAJB1*, encoding the HSP70 cofactor DnaJ homolog subfamily B member 1, a member of the HSP40 family (9). The results here mirrored those of *HSPA1A*: a rapid initial rise from similar levels in EP and LP hMSCs, reaching a maximum an hour into the recovery period, before a return to baseline levels; we also again found that *RT_EP_* < *RT_LP_* (Fig. 3*D*). A pronounced response to heat stress was not detected in levels of *HSPB1*, encoding HSP beta-1, or *HSPA2*, encoding heat shock-related 70 kDa protein 2, in either EP or LP hMSCs (*SI Appendix*, Fig. S3 *F* and *G*). The stress response is understood to be regulated by the transcription factor HSF1 (10). In the absence of stress, HSF1 forms a complex with chaperone proteins located in the cytoplasm. Stress conditions cause this complex to disassemble, releasing HSF1 and enabling it to translocate to the nucleus where it can activate the transcription of stress response proteins. Our data suggests that this mechanism is unaffected by senescence: the rapidity and magnitude of the transcriptional response are similar in LP vs. EP hMSCs. In support of this, quantification of HSF1 protein in the nucleus by IF showed no significant differences between EP and LP hMSCs following heat stress; likewise, *HSF1* transcript showed little response to stress in either EP or LP hMSCs (*SI Appendix*, Fig. S3 *H*–*K*). In summary, senescent hMSCs appeared to misregulate their stress response at a protein level, rather than through an inability to control transcription. We would therefore interpret the slower return of chaperone transcripts to baseline levels in senescent cells following stress as evidence that the burden of unfolded protein persists for longer.

### Loss of Protein Turnover and Translation Machineries Are Limiting Factors in the Stress Response of Senescent Cells.

**Fig. 4.**
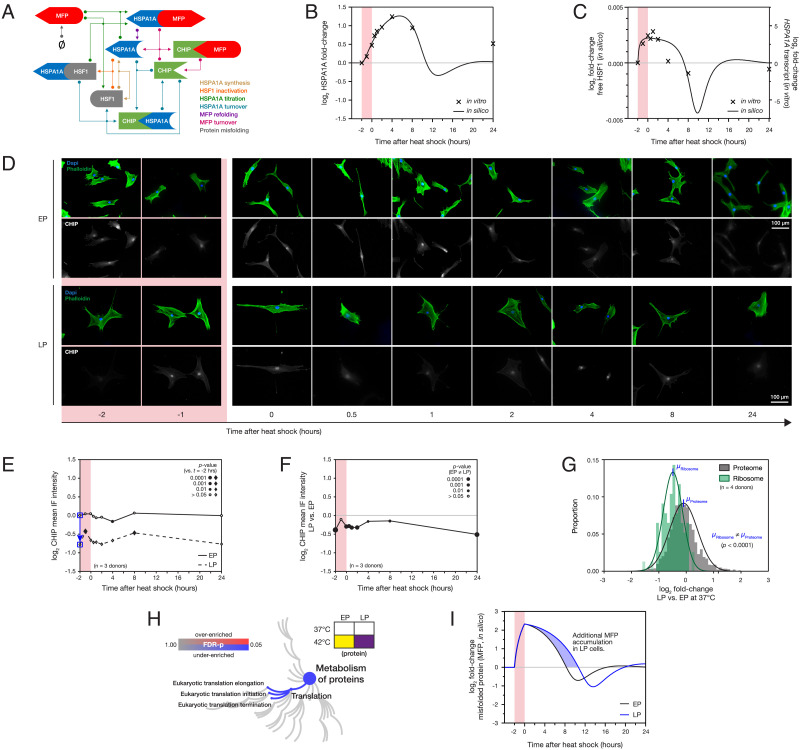
Loss of translational capacity and misfolded protein ubiquitination machinery causes increased proteotoxicity in senescent cells. (*A*) Schematic of the titration model of HSPA1A regulation. Arrows represent processes with rates defined in *SI Appendix*Table S7. (*B*) Ordinary/delayed differential equation (ODE/DDE) model simulation of the stress response. The in silico model was given sufficient time to reach a stable equilibrium, before a proteotoxic stress was simulated by increasing the rate at which misfolded proteins were generated within the model for 120 min. At each time interval, the in silico concentration of HSPA1A was recorded and is shown overlaid with data acquired by immunofluorescence (IF) from early passage (EP) human mesenchymal stem cells (hMSCs) in vitro (Fig. 3*B*). (*C*) As (*B*), but showing concentrations of modeled active HSF1 overlaid, with a scaling factor, onto experimentally-derived *HSPA1A* transcript levels in EP hMSCs (Fig. 3*C*). (*D*) Representative IF images of E3 ubiquitin-protein ligase CHIP in EP and late passage (LP) hMSCs before, during and for 24 h following a 2-h heat shock treatment at 42 °C. (*E*) Quantification of mean CHIP intensities in images shown in panel *D*. Size of points indicates significance of change in protein level vs. preheat shock, with solid points showing *P* < 0.05 (ANOVA, *n* = 3 primary donors). Blue arrow indicates the change in prestress CHIP levels used to modify the ODE/DDE mode. (*F*) Ratios of intensities of CHIP in LP vs. EP hMSCs before, during and after heat shock. Point size indicates significance of where EP ≠ LP, with solid points showing *P* < 0.05 (ANOVA, *n* = 3 primary donors). (*G*) Levels of proteins identified as constitutive of the ribosome (77 proteins) compared to the whole proteome in LP vs. EP hMSCs in the absence of heat shock. Curves show Gaussian fits to the data (with maxima at *µ*_Ribosome_ and *µ*_Proteome_). Levels of ribosomal proteins were supressed relative to the whole proteome in LP vs. EP cells (*P* < 0.0001, two-tailed *t* test). (*H*) Changes to the “Metabolism of proteins” pathway family between stressed EP and LP populations. (*I*) ODE/DDE model simulation of misfolded protein (MFP) levels in the stress response of EP and LP cells.

To investigate how senescence affects the proteotoxic stress response and predicts the consequence of any alterations, we utilized a system of ordinary/delay differential equations (ODE/DDE) to model the concentrations of key factors, using parameters informed by our experimental findings. Building on previous models (45, 46), our model considered interactions between four populations: the chaperone protein HSPA1A, the transcription factor HSF1, the E3 ubiquitin-protein ligase CHIP (an abbreviation of “C-terminus of HSP70 interacting protein”, and protein product of the *STUB1* gene), and intracellular MFP. Reaction rates were derived to represent seven well-defined biological processes between these populations (Fig. 4*A* and *SI Appendix*, Table S7). CHIP was included in the model following our observation that short-term regulation of HSPA1A was principally imposed downstream of transcription. CHIP is known to work with HSP70 family proteins in the HSR but is also a cofactor in HSPA1A turnover (47, 48). CHIP has been shown to ubiquitinate HSPA1A and its client proteins, marking them for turnover but with greater affinity for MFPs than for the chaperone (49). This mechanism reduces the turnover rate of chaperones while the concentration of MFP is high and increases the turnover rate of chaperones during recovery from stress, where MFP concentration is low, but chaperone concentration is elevated. The mean mass spectrometry signal ratio between HSPA1A and CHIP across the four EP prestress biological replicates (*SI Appendix*, Fig. S4*A*) was used in the model as the ratio of concentrations between HSPA1A and CHIP at equilibrium. Practically, this was done by adjusting CHIP levels based on HSPA1A at every time point until a stable equilibrium was reached (*SI Appendix*, Fig. S4*B*). Parameters that could not be inferred from theory, directly from experimental results, or literature evidence ( $k,K_{d},k_{2},k_{4},k_{5},k_{7}$ ) were chosen to optimize the model’s ability to replicate in vitro HSPA1A protein dynamics in EP cells observed in IF data. Several orders of magnitude were trialled for each variable to obtain initial conditions for the optimization (*SI Appendix*, Fig. S4 *C*–*E*).

The model was used to simulate a 2-h heat shock, comparing the dynamics of HSPA1A levels calculated in silico to those acquired experimentally in EP cells (Fig. 4*B*). An initial test of accuracy was performed by comparing in silico and experimental half-lives of HSPA1A: switching off HSPA1A synthesis in the model resulted in a half-life of 100 min (*SI Appendix*, Fig. S4*F*), comparing extremely well with a literature value of 99 min (50). Furthermore, if the model accurately described HSF1 activation dynamics, we would expect it to closely follow the dynamics of *HSPA1A* transcript, with an associated scaling factor. To investigate this relationship, we superimposed active HSF1 concentrations evaluated from in silico modeling with experimentally derived *HSPA1A* mRNA levels in EP cells (Fig. 4*C*). Active HSF1 increased immediately following stress, reaching a plateau after approximately 1 h; active HSF1 then gradually decreased, dropping below the levels observed in the prestress equilibrium state before recovering to initial levels. This behavior was matched by our experimental measurements of *HSPA1A* mRNA and is consistent with reported characterizations of HSF1 activation dynamics in HeLa cells (51). Our experimental analysis was focused on the 10 h following the onset of stress, and our ability to model beyond that period is limited. Cells have been reported to acquire increased thermotolerance following stress (52, 53), but the mechanisms underpinning this change are not well-characterized. Nonetheless, by varying parameters in silico, we were able to analyze sensitivity to each associated process and speculate on the effects of additional mechanisms. Increased rates of HSPA1A synthesis affected the rapidity of the stress response, as well as the persistence of unfolded protein, and damping of the return to equilibrium (*SI Appendix*, Fig. S4*G*). These characteristics of the stress response were also modulated by parameters such as the strength of HSPA1A-client interactions, protein turnover, misfolding and refolding rates, and the rate of HSF1 inactivation (*SI Appendix*, Fig S4 *H*–*M*). Although not explicitly considered by the model, these parameters could be affected in vitro by processes such as post-translational modification, complex formation, and translocation.

Having verified the consistency of our ODE/DDE model with findings in vitro, we sought to investigate how changes observed in senescent cells could affect simulations of the stress response. With our earlier experimental observations of the temporal dynamics of the HSPA1A stress response (Fig. 3 *A*–*C*) supporting the hypothesis that transcription takes hours to dominate HSPA1A regulation, CHIP concentrations in EP and LP cells were analyzed to investigate whether a change in CHIP-mediated turnover could be a limiting factor in the senescent stress response. IF imaging showed a significant decrease in CHIP levels in LP vs. EP cells (Fig. 4*D*; see Fig. 4 *E* and *F* for significance values, determined by ANOVA). In contrast, senescence had a minimal effect on levels of transcription factor HSF1 (*SI Appendix*, Fig. S3*G*). Following our finding that the limiting factor in the stress response of LP cells was downstream of chaperone transcription, we compared the relative quantification of the levels of ribosomal proteins in our EP vs. LP mass spectrometry data. A comparison of the 77 detected ribosomal proteins showed a significant decrease in ribosomal machinery in senescence (a mean log_2_ fold-change of −0.4873; *P* < 0.0001, two-tailed *t* test; Fig. 4*G*), suggestive of a loss of translational capacity. This was matched with suppression of associated GO terms according to an FDR-corrected Protein Analysis Through Evolutionary Relationships (PANTHER) (31) analysis of differentially abundant proteins showing underenrichment of protein metabolism, including translation initiation, elongation, and termination (*P* < 0.05; Fig. 4*H*). In addition to weakened chaperone activity, previous reports have suggested that a decline in the ubiquitin-proteasome system and autophagy pathways can contribute to protein aggregation (54, 55). However, analysis of our proteomics data showed no overall loss of proteasomal machinery in LP vs. EP hMSCs, relative to the overall proteome (FDR-corrected *P* = 0.99; *SI Appendix*, Fig. S4*N*).

### In Silico Modeling Predicts that Senescent Cells Are More Susceptible to an Accumulation of Proteotoxic Damage.

Our experimental observations of senescence-induced loss of CHIP and translational capacity were incorporated into our ODE/DDE model by i) multiplying the prestress CHIP concentration by a factor of $2^{-0.7799}=0.5824$ (reflecting data indicated by the blue arrow on Fig. 4*E*) and ii) multiplying the parameter k—representing the maximal rate of HSPA1A synthesis—by a factor of $2^{-0.4873}=0.7134$ (from proteomic quantification of ribosomal components, *µ*_Ribosome_ in Fig. 4*G*). In silico, these alterations lead to a decrease in the magnitude of HSPA1A upregulation in response to stress, and an extension of the time taken for transcriptionally active HSF1 to return to prestress levels (*SI Appendix*, Fig. S4 *O* and *P*)—both consistent with our in vitro quantification of HSPA1A protein and transcript in the stress responses of LP cells. The model enabled us to estimate the increased burden of MFPs in senescent cells subjected to heat stress—a quantity difficult to measure directly by experimental means (Fig. 4*I*): the period of proteotoxic stress was prolonged in senescent cells (from 10.3 to 12.7 h), and the area under the MFP curve (i.e., persistence of MFP in the cell) increased by approximately 30%.

### In Vitro Assays Confirm that Senescent Cells Are More Prone to Stress-Induced Changes in Protein Aggregation and Folding State.

**Fig. 5.**
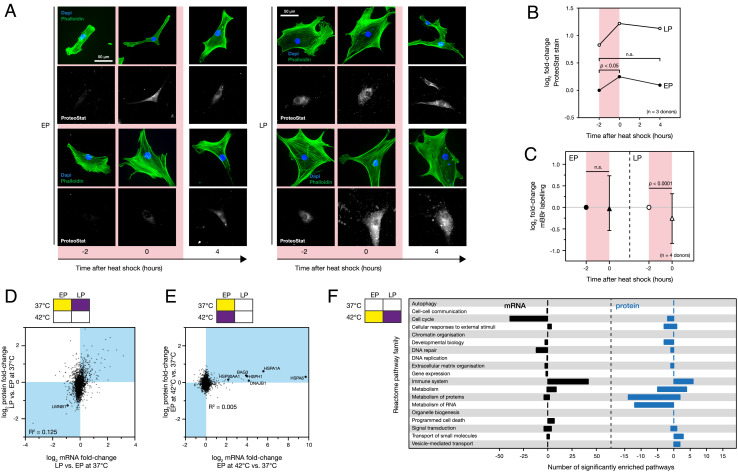
Senescence-induced suppression of the stress response increases protein aggregation; senescence affects transcriptional regulation. (*A*) Representative images of early passage (EP) and late passage (LP) human mesenchymal stem cells (hMSCs) with ProteoStat staining against protein aggregates, immediately before, immediately after, and 4 hours following a 2-hour heat shock treatment at 42 °C. (*B*) Quantification of Proteostat staining: EP hMSCs showed a significant increase in protein aggregation during the 2-hour heat shock treatment, which returned to baseline levels 4 hours later; levels of protein aggregation in LP cells remained elevated throughout the treatment and recovery period (ANOVA, *n* = 3 primary donors, *SI* Fig. S5A). (*C*) The distribution (median and interquartile range) of abundance changes of 359 monobromobimane (mBBr)-tagged peptides from 252 proteins in response to stress in EP and LP hMSCs subjected to 2-hour heat treatment at 42 °C. Significance calculated from non-parametric Wilcoxon signed-rank test, *n* = 4 primary donors. (*D*) Correlation between transcript and protein changes in LP vs. EP hMSCs (R^2^ = 0.125). (*E*) Plot showing relationship between transcript and protein changes in EP hMSCs subjected to 2-hour heat shock treatment at 42 °C vs. an unstressed control (R^2^ = 0.005). Chaperone-associated genes were substantially upregulated, with corresponding smaller increases in protein products. Volcano plots showing significance of changes in transcript levels in EP and LP hMSCs subjected to heat stress can be found in *SI* Figs. S5C-F. (*F*) Selected results of PANTHER statistical enrichment analysis of the fold-changes between the stress transcriptomes of EP and LP hMSCs (59). 141 pathways were significantly enriched across the 12 top-tier Reactome pathways, as illustrated in Fig. 1E. Positive values indicate over-enriched pathways in LP hMSCs, while negative values indicate under-enriched pathways. *n* = 5 primary donors for all transcript data.

We tested for increased protein aggregation using the ProteoStat dye, which has increased fluorescence when its rotational freedom is constrained during binding to aggregates (56). Donor-matched EP and LP hMSCs were imaged before, immediately after, and 4 h following a 2-h treatment at 42 °C (Fig. 5*A*). Quantification of ProteoStat fluorescence showed that protein aggregation was significantly increased immediately following heat stress in EP hMSCs (*P* = 0.04, ANOVA) but was recovered after 4 h (Fig. 5*B* and *SI Appendix*, Fig. S5*A*). By contrast, in LP hMSCs levels of ProteoStat staining were elevated and remained so immediately and 4 h following heat treatment. We performed an orthogonal test for the presence of altered folding states using mass spectrometry in conjunction with monobromobimane (mBBr) protein labeling (57, 58). mBBr selectively labels reduced cysteine residues typically buried in the hydrophobic cores of folded proteins; however, reactivity can be modulated by protein unfolding (increasing access of mBBr to cysteine residues) or aggregation (decreasing access). As such, modulated levels of mBBr-adduct peptides quantified by LC-MS/MS, in comparison to a control state, can be interpreted as a change to the folding-state of the proteome. Here, a broadening of the distribution of abundances of mBBr-adduct peptides indicated protein conformational changes in response to thermal stress (*SI Appendix*, Fig. S5*B*). Fig. 5*C* shows mean log_2_ fold-changes to 359 unique mBBr-adduct peptides from 252 proteins detected in EP and LP hMSCs subjected to a 2-h treatment at 42 °C. Consistent with the ProteoStat staining and our in silico prediction that proteostasis would be perturbed to a greater extent in LP cells under heat stress, a significant change was observed in the mBBr-labeling profiles of LP cells (*P* < 0.0001, *t* test), but no significant change was found in EP cells. Together, a combination of in silico and in vitro experiments have shown that a consequence of an abrogated stress response is that senescent cells are more susceptible to protein misfolding and the accumulation of protein aggregates.

### Transcriptomic Profiling Suggests Wider Consequences of a Senescence-Dampened Response to Stimulation.

To better understand the broader consequences of senescence on the stress response, we reexamined the conditions of our initial proteomic experiment (Fig. 1) using transcriptomic profiling (*SI Appendix*, Fig. S5 *C*–*F*). This identified 5% of 15,782 transcripts that were significantly altered in LP vs. EP hMSCs in the absence of stress (262 downregulated, 471 upregulated; FDR-corrected ANOVA; *SI Appendix*, Fig. S5*C*). The effects of senescence on mRNA and product-protein levels showed only a modest positive correlation (R^2^ = 0.125; Fig. 5*D*) with heat stress having a less marked effect on the transcriptome than the intrinsic differences between EP and LP cells (*SI Appendix*, Fig. S5*D*). The same trend was also noted for the proteome. Similarly, an even weaker correlation was noted between the protein and transcript levels following heating (R^2^ = 0.005; Fig. 5*E*) compared to that due to senescence, consistent with a greater degree of protein-level regulation in the stress response.

In another parallel to our earlier proteomic data, the transcriptomic response to heat treatment was dampened in LP vs. EP cells: 2.8% of transcripts were significantly affected when LP hMSCs were subjected to a 2-h treatment at 42 °C, relative to a control (98 downregulated, 367 upregulated; FDR-corrected ANOVA; *SI Appendix*, Fig. S5*E*). Nonetheless, a substantial overlap was seen between transcripts affected by heat stress in EP and LP cells, with 295 upregulated and 53 downregulated transcripts common to both datasets. Application of our chaperome network analysis to the transcript data showed no significant differences in chaperome modules in LP vs. EP cells in the absence of stress (*SI Appendix*, Fig. S5*G*), but modules were broadly upregulated following heat treatment irrespective of passage (*SI Appendix*, Fig. S5 *H* and *I*); a transcriptional response to stress is consistent with previous observations reported in lung fibroblast cells (59). Of note, the transcriptional response to stress was significantly greater in HSP70 and ER modules in LP hMSCs (*P* < 0.05, ANOVA; SI *SI Appendix*, Fig. S5*J*). However, many HSP70 module components were commonly upregulated in the stress responses of both EP and LP cells, including *HSPA6*, *HSPA7*, and *HSPA1A*. This overlap between the transcriptomic response to stress in EP and LP cells had not been observed at the protein level. Pathways associated with the cellular responses to external stimuli were upregulated at the transcript level in the LP vs. EP response to stress (*SI Appendix*, Fig. S5*K*), where we had previously seen them downregulated at the protein level (Fig. 1*F*). This disparity shows that while EP and LP cells may attempt to carry out a similar stress response, the actual response achieved at protein level differs greatly. This was seen more systematically by contrasting the correlation between the transcriptomic response to heat stress in LP vs. EP hMSCs (R^2^ = 0.463; *SI Appendix*, Fig. S5*L*) and the same comparison at the protein level (R^2^ = 0.005; *SI Appendix*, Fig. S5*M*). Interestingly, however, the senescence-associated suppression of translational machinery observed in the protein-level pathway analysis (Fig. 4*H*) was mirrored at transcript level (*SI Appendix*, Fig. S5*N*).

A systematic PANTHER enrichment analysis of Reactome pathways applied to both our proteomic and transcriptomic datasets identified significant enrichment of 141 pathways at mRNA level and 61 pathways at protein level (*p* < 0.05; Fig. 5*F*) (31). In the transcriptional analysis, 39 cell cycle pathways were detected as underenriched in LP populations as well as 2 pathways in the protein analysis, with no cell cycle pathways overenriched. This is consistent with LP hMSC populations containing high numbers of senescent cells which have undergone cell cycle arrest. Strikingly, 42 transcriptional and 6 protein pathways associated with the immune system were significantly overenriched in LP populations following stress, compared with EP populations. This is consistent with “senescence hallmarks”—inflammation and fibrosis—reported in muscle (60), and potentially has consequences on the immunosuppressive properties of hMSCs that are key to their application in regenerative medicine.

## Discussion

### Cellular Resilience May Be Key to the Success of Regenerative Medicine Strategies.

Therapeutic applications of hMSCs are commonly based on an “autologous” strategy in which cells are extracted from the patient, expanded by culture in vitro, before being implanted back into damaged tissues. Once implanted, hMSCs are thought to be capable of migration to sites of injury and to improve tissue health through paracrine signaling (22, 61). A recent review of over 350 clinical trials involving bone marrow-derived hMSCs (with phase 3 trials most commonly addressing graft-vs.-host disease, GvHD, and neurological, joint, and cardiovascular conditions) found that trials utilizing small doses of cells were most likely to be ineffective (62). The authors highlighted the need for extended in vitro expansion prior to treatment but also noted a lack of optimization and standardization across culture protocols. Similarly, a review of 15 clinically approved hMSC treatments found no information on passage number was given for any of the treatments, exemplifying the incomplete understanding of how hMSC expansion affects their therapeutic performance (63). Increased time in culture induces cellular senescence through telomere shortening, a consequence of increased likelihood in cells sourced from elderly patients. In senescent hMSCs, the paracrine signaling which promotes tissue repair is replaced with a senescence-associated secretory phenotype (SASP) which correlates with protein markers of aging (64). Consisting of inflammatory, extracellular matrix-modifying, and tumorigenic factors, the SASP has been shown to induce senescence in exposed healthy cells (65, 66) and be detrimental to regeneration (60). Therefore, while pharmacological methods of senescent cell clearance are in development (67), the harmful effect senescent cells have on surrounding tissues cannot be neglected.

Tissue characteristics associated with cell transplantation, including infection and inflammation, have been noted as potential triggers of the cellular HSR (25). Treatment efficacy has been shown to depend on the resilience of hMSCs to the post-transplant environment and demonstrated that cultures from aged human donors, which contained a higher proportion of senescent cells and had increased incidence of cell death when exposed to a hypoxic environment reflective of the post-transplant environment in ischemic patients (68). In the LP vs. EP response to thermal stress (Fig. 5*F*), we have shown an overenrichment of transcriptional pathways associated with programmed cell death; underenrichment in transcriptional and proteomic pathways associated with DNA repair; and underenrichment in proteomic pathways associated with cellular responses to external stimuli, despite overenrichment at the transcriptional level. As shown in Fig. 1*F*, the top-tier Reactome pathway “cellular responses to external stimuli” and the sub-pathway “cellular responses to stress” were both significantly underenriched (FDR-corrected *P* values of 0.026 and 0.015, respectively) in proteomic data from LP populations following stress, compared to EP. Fig. 5*E* demonstrated that this attenuation was not caused by a lack of transcription of stress response proteins or upstream thermosensing. On the contrary, transcripts of several stress response factors were elevated in LP populations compared to EP, underscoring the fact that the stress response was compromised primarily at the *protein* level in senescent cells. The success of trials of allogeneic treatments, such as described by Marx et al. (69), has highlighted that the ability to modulate the immune system is a key feature of the therapeutic action of MSCs. Studies conducted in vitro have shown hMSCs to suppress lymphocyte proliferation (70); an antibacterial effect has also been demonstrated to prevent infection (71). These features have led to clinical trials using hMSCs to ameliorate the effects of GvHD (72). Our data have implications for such approaches, as we noted that Reactome pathways associated with the immune system were compromised in the stress response of LP vs. EP hMSCs, in both transcripts and protein products (Fig. 5*F*).

Although this study has focused on the basic cell biology of senescence, it highlights important points to consider in medical applications of MSCs: that in vitro expansion of MSCs may be detrimental to their efficacy in the clinic as replication-induced senescence impairs cellular function and resilience; furthermore, an understanding of the role of proteostasis in this process must consider protein as well as transcriptional dysregulation. Lastly, while here we have considered intracellular effects over a limited time span, the negative consequences of senescence—either from aging or cell implantation—may be systematic and chronic, modulated by SASP products and immunomodulatory signaling.

### In Silico Tools Can Effectively Compliment In Vitro Studies of the Stress Response.

Mathematical models of biological processes have increasingly been used not only to validate hypotheses but also as means to generate new hypotheses (73). Biological processes are complex, and a functional descriptive model can demonstrate a working understanding of a process. Going further, one can perturb single elements of a system in silico, or make measurements or predictions about system features that would be difficult to measure directly via experiment. Here, we created an ODE/DDE model of the stress response with the aim—when informed by temporally resolved experimental data—of challenging the current understanding of HSPA1A regulation. Models attempting to describe this process have thus far been built around the theory of mass synthesis of an already highly abundant protein and have generally neglected post-translational regulation (45, 46, 74–79). Our model has built on the minimal models developed by Sivery et al. and Zheng et al. The former of these models assumed an unchanged rate of HSPA1A turnover during stress, while the latter disregarded HSPA1A turnover altogether. Both designs therefore neglected evidence of upregulated HSPA1A half-life during stress (49, 50, 80). Moreover, predominantly stabilizing chaperone machinery under stress, rather than synthesizing additional protein, is supported in the literature (50, 81). This suggests an important general point: that when faced with a need to rapidly remodel protein machinery in order to address an acute or short-term demand, particularly in high number-density proteins such as those of the chaperome or cytoskeleton, cells may draw on other tools besides an upregulation of transcription. It may in many cases be faster, and more efficient in terms of conserving cellular resources, to regulate post-translational modifications and turnover rates (and potentially also location within the cell, as evidenced by our microscopy data). Our in silico simulations have predicted that senescent-associated loss of translational and ubiquitin-ligase capacities leads to a greater accumulation of MFPs during proteotoxic stress. These factors may be of key importance to understanding the consequences of cellular senescence, and by extension how the therapeutic efficacy of MSCs might be improved, as well as broader facets of the aging process and age-associated disease.

## Materials and Methods

All procedures and experiments were performed with approval from the NHS Health Research Authority National Research Ethics Service (approval number 10/H1013/27) and the University of Manchester. Bone marrow tissue was obtained with full written informed consent from donors undergoing knee or hip replacement surgery. No patient-identifiable information was acquired. Comparisons were made between donor-matched proliferating and senescent primary hMSCs, under control conditions and when subjected to a 2-h heat shock treatment at 42 °C and following a 24-h recovery period. Details of heat shock and inhibitor treatments, cell imaging, transcript and protein quantification, proteome modularity analysis, and a kinetic model of the stress response can be found in *SI Appendix*.

## Supplementary Material

Appendix 01 (PDF)

## Data Availability

Proteomics data have been deposited to the ProteomeXchange Consortium via the PRIDE partner repository; transcript data to EMBL-EBI ArrayExpress. PXD025280, EP and LP hMSCs subjected to heat shock (82); PXD025305, EP and LP hMSCs subjected to heat shock in combination with HSPA1A inhibition (83); PXD027056, EP hMSCs subjected to HSP90 inhibition (84); PXD025329, monobromobimane labelling of EP and LP hMSCs subjected to heat stress (85). RNA-Seq data is available with identifier E-MTAB-10415.
