# Supplementary material for: Loss of regulation of protein synthesis and turnover underpins an attenuated stress response in senescent human mesenchymal stem cells
Source: Proc Natl Acad Sci U S A. 2023 Mar 29;120(14):e2210745120. doi: 10.1073/pnas.2210745120 (PMC10083568; doi:10.1073/pnas.2210745120)
Supplement: Supplementary file 1 — Appendix 01 (PDF) [file pnas.2210745120.sapp.pdf]

SUPPLEMENTAL INFORMATION

Loss of regulation of protein synthesis and turnover underpins an attenuated stress response in senescent human mesenchymal stem cells

Jack Llewellyn[1, 2], Venkatesh Mallikarjun[1, 2], Ellen Appleton[1, 2], Maria Osipova[1, 2], Hamish TJ Gilbert[1, 2, 3], Stephen M Richardson[2], Simon J Hubbard[4, 5] and Joe Swift[1, 2, 5]

(1) Wellcome Centre for Cell-Matrix Research, Oxford Road, Manchester, M13 9PT, UK.

(2) Division of Cell Matrix Biology and Regenerative Medicine, School of Biological Sciences, Faculty of Biology, Medicine and Health, Manchester Academic Health Science Centre, University of Manchester, Manchester, M13 9PL, UK.

(3) Present address: School of Life Sciences, Faculty of Natural Sciences, Keele University, Newcastle-under-Lyme, ST5 5DX, UK.

(4) Division of Evolution and Genomic Sciences, School of Biological Sciences, Faculty of Biology, Medicine and Health, Manchester Academic Health Science Centre, University of Manchester, Manchester, M13 9PL, UK.

(5) Correspondence to SJH (simon.hubbard@manchester.ac.uk) or JS (joe.swift@manchester.ac.uk).

CONTENTS

Supplemental discussion of chaperone inhibition experiments … … … … 3

Supplemental materials and methods … … … … … … … 5

Supplemental figures S1 – S6 … … … … … … … … 16

Supplemental table S7 … … … … … … … … 27

Supplemental references … … … … … … … … 28

SUPPLEMENTAL DISCUSSION OF CHAPERONE INHIBITION EXPERIMENTS

Early passage cells are resistant to inhibition of chaperone activity in the absence of thermal stress.

In order to test the interconnectedness of the chaperone network shown in Figure 2A, we examined regulation of the proteome in cells subjected to targeted inhibition of chaperone machinery. The small molecule 2-phenylethynesulfonamide (PES) has been shown to bind selectively to HSPA1A, inhibiting its activity by preventing interactions with its cochaperones (1-4). We found that treatment of EP hMSCs with PES did not greatly perturb the proteome: of 1830 proteins detected with ≥3 peptides-per-protein, only 26 proteins were significantly increased, and 22 decreased ($p < 0.05$, FDR-corrected ANOVA; SI Fig. S6A). The five major chaperome modules identified in Figure 2A were also not significantly affected (ANOVA, SI Fig. S6B), suggesting that the system was resistant to PES treatment in the absence of additional stress factors. Nonetheless, we did identify proteins within the HSP70 module that were possibly upregulated as part of a compensatory mechanism, such as heat shock 70 kDa protein 6 (HSPA6) and ER chaperone BiP (HSPA5) (SI Fig. S6C).

To test the generality of this apparent resistance to perturbation, we also examined the effects of targeted inhibition of HSP90 machinery. Gedunin and radicicol both affect HSP90 regulation: gedunin by disrupting interaction with the HSP90-cochaperone prostaglandin E synthase 3 (PTGES3) (5), and radicicol by binding to the N-terminal nucleotide-binding domain of HSP90 proteins, preventing critical ATPase activity (6). Treatment of EP hMSCs with gedunin broadly perturbed the proteome (of 2181 proteins detected with ≥3 peptides-per-protein, 223 were significantly increased and 428 decreased; SI Fig. S6D), but there was no overall significant change to chaperone modules (ANOVA; SI Figs. S6E, F). Treatment with radicicol had a narrower effect on the proteome (129 significantly increased and 92 decreased proteins; SI Fig. S6G), and the ER chaperone module was significantly increased ($p = 0.009$, ANOVA; SI Fig. S6H). In similarity with the response to HSP70 inhibition, neither of the HSP90 inhibitors caused a significant overall perturbation to their target chaperome module (i.e. HSP90 machinery; SI Figs. S6F, I). We did, however, observe shared changes in the proteomes of cells treated with either HSP90 inhibitor (SI Fig. S6J), and common changes within the HSP90 module consistent with a compensatory response: both drug treatments resulted in increased levels of inducible (HSP90AA1), constitutively-expressed (HSP90AB1) and ER-resident (HSP90B1) forms of HSP90.

Senescence increases sensitivity to heat stress when combined with inhibition of HSP70. Having found EP hMSCs to be robust to inhibition of key chaperone proteins in the absence of stress, we applied the same proteomic tools to examine the effects of combining HSP70 inhibition with heat shock and senescence. Analysis of the effect of PES treatment on EP hMSCs subjected to a 2-hour treatment at 42 °C showed a broader perturbation to the proteome than PES-treated cells maintained at 37 °C (comparing SI Fig. 6K to SI Fig. S6A): 89 proteins were significantly increased, and 23 decreased ($p < 0.05$, FDR-corrected ANOVA). The chaperome module related to HSP70 machinery was significantly upregulated ($p = 0.004$, ANOVA; SI Fig. S6L), and quantities of individual protein components within the module were generally increased (SI Fig. S6M). This result mirrors the response of EP hMSCs to heat stress in the absence of PES (Figs. 2D, E), interpretable as the cells responding to proteotoxic stress, finding the response ineffective due to the inhibition of HSP70, and therefore increasing its magnitude. A comparison between LP hMSCs with and without PES treatment, in the absence of heat stress, showed a muted response. Only 24 proteins were significantly increased and 14 decreased ($p < 0.05$, FDR-corrected ANOVA; SI Fig. S6N), and the HSP70 chaperome module was not significantly affected (SI Figs. S6O, P). This is similar to the result found in EP cells (SI Figs. S6A-C), as in the absence of stress, inhibition of specific stress response machinery seemed to have a limited impact. In contrast, the effect of PES treatment on senescent hMSCs subjected to a 2-hour treatment at 42 °C was more substantial: 92 proteins were significantly increased, and 125 decreased ($p < 0.05$, FDR-corrected ANOVA; SI Fig. S6Q). However, unlike in EP cells, this perturbation was not reflected in a significant perturbation to the HSP70 chaperome module (SI Fig. S6R), and many of the individual proteins within the module appeared to be suppressed (SI Fig. S6S). This is again reminiscent of experiments performed in the absence of PES, where LP cells were unable to mount a chaperome response to thermal stress (Fig. 2F). Interestingly, PES treatments on LP cells at both temperatures caused an upregulation of the CCT/TRiC chaperome module (SI Figs. S6O, R), notably suppressed in senescence (Fig. 2D). This hints at crosstalk between chaperome modules, and a possible role for HSP70 machinery in cytoskeletal maintenance. In addition to demonstrating the application of our network analysis with targeted inhibition of the chaperone machinery by known inhibitors, these results highlight the failure of senescent cells to respond to proteotoxic stress in the same way as early passage cells. Furthermore, this dysregulation was not caused by a desensitization to temperature – the proteome of LP cells was more widely affected by PES treatment at elevated temperature – but rather a failure to remodel specific features of the chaperome.

SUPPLEMENTAL MATERIALS AND METHODS

Primary cell culture. Human mesenchymal stem cells (hMSCs) were isolated from bone marrow using established methodology (7). The only information associated with each donor sample was source site, donor sex and age; no patient-identifiable information was obtained. hMSCs were cultured on tissue culture treated polystyrene (TCTP) in low-glucose DMEM with pyruvate (Thermo Fisher Scientific) supplemented with 10% fetal bovine serum (FBS, Labtech.com) and 1% penicillin/streptomycin cocktail (PS, Sigma-Aldrich). All quantitative comparisons were made between hMSCs from the same donor, matched at early passage ("EP", proliferating) and late passage ("LP", senescent): a portion of cells were kept in frozen storage whilst donor-paired cells were passaged to a point of replicative senescence, allowing LP vs. EP experiments to be performed at the same time. hMSCs from different donors were not pooled and *n* numbers quoted in the paper refer to biological replicates (i.e., from different donors). Details of individual donors, the passage numbers at which cells were used, and in which experiments, are summarised in the following table.

Donor ID	Donor gender	Source site	Donor age	Passage number (proliferating)	Passage number (senescent)	Experiments using these cells
TH112	F	Hip	74	6	11	• β-galactosidase staining
WH143	M	Hip	58	2	7	• Mass spectrometry (mBBr)
WH176	F	Hip	64	2	8	• Cell morphology<br>• Immunofluorescence (CHIP)<br>• Mass spectrometry (HSP90 inhibition)<br>• RNA sequencing
TH191	F	Hip	61	4	9	• β-galactosidase staining<br>• RT-qPCR
TH194	M	Knee	65	3	8	• β-galactosidase staining<br>• Cell morphology<br>• Immunofluorescence (CHIP)<br>• Proteostat staining<br>• Mass spectrometry (HSP90 inhibition)<br>• Mass spectrometry (mBBr)

Donor ID	Donor gender	Source site	Donor age	Passage number (proliferating)	Passage number (senescent)	Experiments using these cells
WH211	F	Hip	36	7	18	• Cell morphology<br>• Immunofluorescence (HSP70 and HSF1)<br>• Proteostat staining<br>• RT-qPCR<br>• Mass spectrometry (heat stress; HSP70 inhibition)
TH215	M	Knee	73	3	5	• Cell morphology<br>• Immunofluorescence (CHIP)<br>• Mass spectrometry (heat stress; HSP70 inhibition)<br>• Mass spectrometry (mBBr)
TH223	M	Knee	64	4	7	• Mass spectrometry (HSP90 inhibition)<br>• RNA sequencing
WH226	F	Hip	54	2	9	• Cell morphology<br>• Immunofluorescence (HSP70 and HSF1)<br>• Mass spectrometry (heat stress; HSP70 inhibition)
WH230	M	Hip	43	3	7	• Cell morphology<br>• Immunofluorescence (HSP70 and HSF1)<br>• Proteostat staining<br>• RT-qPCR
TH269	M	Knee	70	1	7	• Mass spectrometry (heat stress; HSP70 inhibition)
TH270	M	Knee	72	1	7	• RNA sequencing
TH296	M	Hip	68	2	6	• RT-qPCR<br>• RNA sequencing
TH305	M	Knee	67	3	7	• RNA sequencing
TH309	F	Knee	69	4	10	• Mass spectrometry (mBBr)

Heat shock and inhibitor treatments. For immunofluorescence (IF) assays, hMSCs were seeded at a density of 500 cells/cm^2 onto #1.5-thickness coverslips (SLS) in 35 mm petri dishes (Corning) for 24 h prior to experimentation. For proteomic and transcript assays, hMSCs were seeded at a density of 7000 cells/cm^2 into T75 flasks (Corning) 24 h prior to experimentation. Cells were incubated for 2 h at 42 °C in a 5% CO_2 humidified incubator, while control groups were maintained at 37 °C. For IF imaging and RT-qPCR, nine time points were taken across the 2 h heat shock (HS) and 24 h recovery period: pre-HS; 1 h into HS; immediately following HS; 30 min post-HS; and 1, 2, 4, 8 and 8 h post-HS. In experiments with HSP70 inhibition, media was replaced with fresh media containing 0.01% DMSO (Sigma) and 10 µM 2-phenylethynesulfonamide (PES, Sigma) 30 min prior to HS; comparisons were made to vehicle-only controls. HSP90 inhibitors were used at concentrations of 100 nM for gedunin (Cambridge Bioscience) (8) and 1 µM for radicicol (Sigma) (9), delivered in fresh media containing 0.01% DMSO; cells were treated for 24 h, before comparison to vehicle-only controls.

Immunofluorescence (IF), microscopy and image analysis. Human MSCs were fixed with 4% paraformaldehyde (PFA, VWR International) in deionized (DI) water for 10 min at 37 °C, followed by washing in Dulbecco's phosphate-buffered saline (PBS, Sigma). Cells were permeabilized in 1% Triton-X (Sigma-Aldrich) in PBS for 10 min and blocked with 2% bovine serum albumin (BSA, Sigma-Aldrich), 0.1% Triton-X in PBS at 37 °C for 1 h. Samples were incubated with a monoclonal antibody raised in rabbit against HSPA1A (1:1000; Abcam, ab181606) for 1 h at 37 °C, followed by 4 x washes in PBS. The secondary AlexaFluor-594 donkey anti-rabbit (1:1000; ThermoFisher Scientific, A21207) was added with DAPI (1:500; Sigma Aldrich, D9542) and AlexaFluor-488 Phalloidin (1:500; Cell Signaling Technology, #8878), incubated for 1 h and washed 5 x times with PBS. Coverslips were rinsed 2 x in DI water before mounting onto 1 mm-thick glass slides (Thermo Scientific) using anti-fade mounting medium (Dako). β-galactosidase staining (Cell Signalling Technology) and the Proteostat Aggresome detection system (Enzo Life Sciences Inc.) were used according to the manufacturers' instructions.

Images were collected on a Zeiss Axioimager.D2 upright microscope using 10x and 20x / 0.5 EC Plan Neofluar objective lenses and captured using a Coolsnap HQ2 camera (Photometrics) with Micro-Manager software (version 1.4.23). Band pass filter sets for DAPI, FITC and Texas red were used to prevent bleed between channels. Images were processed using Fiji and ImageJ (version 2.0.0, National Institutes of Health, USA); CellProfiler (version 2.1.1, Broad Institute, USA) (10) was used to quantify cell morphometric parameters. Cell aspect ratio was defined as being between the lengths of long and short sides of a minimally-sized box bounding each cell. Images were corrected for background fluorescence by subtracting the mean intensity/pixel of a cell-free area from each pixel; all images under comparison in the same experiment had matched exposure and contrast settings.

RT-qPCR. hMSCs were harvested with EDTA-trypsin (Sigma) and pelleted by centrifugation. RNA was extracted from cell pellets using the RNeasy Mini kit (Qiagen), as per the manufacturer's instructions, and its concentration was measured using a NanoDrop 2000 spectrophotometer (Thermo Fisher). 1 µg of mRNA per sample was reverse transcribed using the High Capacity RNA-to-cDNA Kit (ThermoFisher Scientific) in a Verity Thermal Cycler (Applied Biosystems). RT-qPCR was performed in triplicate using SYBR Select Master Mix (ThermoFisher Scientific) using a StepOnePlus Real-Time PCR System (ThermoFisher Scientific). Data was analysed using the $2^{-\Delta\Delta Ct}$ method (11) and normalised to the housekeeping gene *PPIA*. Custom designed and validated primers (PrimerDesign Ltd) were used as follows:

Peptidyl-prolyl isomerase A (*PPIA*)	sense: ATGCTGGACCCAACACAAA
	anti-sense: TTTCACTTTGCCAAACACCA
Heat shock 70 kDa protein 1A (*HSPA1A*)	sense: CCACCAAGCAGACGCAGAT
	anti-sense: CCCTCTCGCCCTCGTACA
Heat shock factor protein 1 (*HSF1*)	sense: CCACCTCCACCCCTGAAAA
	anti-sense: GGAGTCCATAGCATCCAAGTG
Lamin-B1 (*LMNB1*)	sense: CCTTCTTCCCGTGTGACAGTA
	anti-sense: CTACTCGCCTCTGATTCTTCCA
DnaJ homolog subfamily B member 1 (*DNAJB1*)	sense: AATCTCCCACAAGCGGCTAA
	anti-sense: GGTCTGGTCTCCTTCCTTGG

Mass spectrometry (MS) sample preparation and analysis. hMSCs were washed with PBS, harvested with EDTA-trypsin (Sigma), pelleted by centrifugation, re-suspended in PBS and re-pelleted into 1.5 mL LoBind tubes (Eppendorf). Six 1.6 mm steel beads (Next Advance) were added to the cell pellet tube with 30 µL SL-DOC (1.1% sodium dodecyl sulfate (Sigma), 0.3% sodium deoxycholate (Sigma), 25 mM ammonium bicarbonate (AB, Fluka), 0.1% protease inhibitor cocktail set 1 (Calbiochem) and 0.1% phosphatase inhibitor no. 3 (Sigma) in de-ionised (DI) water). Cells were homogenized in a Bullet Blender (Next Advance) at maximum speed for 2 min. Homogenates were cleared by centrifugation (20k g, 5 min). Protein concentrations were measured by Direct Detect spectrophotometer (Millipore). Immobilized-trypsin beads (Perfinity Biosciences) were suspended in 200 µL of digest buffer (1 mM $CaCl_2$ (Sigma) in 25 mM AB) containing 50 µg of lysate protein and shaken overnight in a thermomixer (Eppendorf; 1400 rpm at 37 °C).

The resulting digest was reduced (addition of 4 µL x 0.5 M dithiothretol (Sigma) in 25 mM AB; 10 min shaking at 60 °C) and alkylated (addition of 12 µL x 0.5 M iodoacetamide (Sigma) in 25 mM AB; 30 min shaking in the dark at room temperature). Peptides were acidified by addition of 5 µL x 10% trifluoroacetic acid (Riedel-de Haën) in DI water, and cleaned by two-phase extraction (2 x addition of 200 µL ethyl acetate (Sigma) followed by vortexing, centrifugation and aspiration of the organic layer). Peptides were desalted using POROS R3 beads (Thermo Fisher), in accordance with the manufacturer's protocol, and lyophilized. Peptide concentrations (measured by Direct Detect spectrophotometer, Millipore) in injection buffer (5% HPLC grade acetonitrile (ACN, Fisher Scientific) 0.1% trifluoroacetic acid in DI water) were adjusted to 0.3 g/L.

Samples were analysed by LC-MS/MS using an UltiMate® 3000 RSLC (Dionex Corporation) coupled to a Q Exactive HF (Thermo Fisher Scientific) mass spectrometer. Peptides were separated using a 75 mm × 250 µm inner diameter 1.7 µM CSH C18 analytical column (Waters) with a gradient from 95% A (0.1% formic acid, FA, Sigma) and 5% B (0.1% FA in ACN) to 7% B at 1 min, 18% B at 58 min, 27% B in 72 min, and 60% B at 74 minutes at 300 nL/min. Peptides were selected for fragmentation automatically by data-dependent analysis.

Proteomics data processing. Alignment and peak-picking were performed in Progenesis QI (Waters) and searched using Mascot (Matrix Science UK), against the SWISS-Prot and TREMBL human databases. The peptide database was modified to search for alkylated cysteine residues (monoisotopic mass change, 57.021 Da), oxidized methionine (15.995 Da), hydroxylation of asparagine, aspartic acid, proline or lysine (15.995 Da) and phosphorylation of serine, tyrosine, threonine, histidine or aspartate (79.966 Da). A maximum of 2 missed cleavages was allowed; peptides with charges above +4 or fewer than two isotopes were removed. Peptide detection intensities were exported from Progenesis QI as Excel spreadsheets (Microsoft) for further processing.

Proteomics datasets were analysed using code written in-house in MATLAB with the bioinformatics toolbox (R2015a, The MathWorks, USA). Raw ion intensities from peptides from proteins with fewer than 3 unique peptides per protein were excluded from quantification. Peptide lists were filtered leaving only those peptides with a Mascot score corresponding to a Benjamini-Hochberg false discovery rate (BH-FDR) (12) of < 0.2. Normalisation was performed as follows: raw peptide ion intensities were log-transformed to ensure a normal distribution and normalised within-sample by equalising sample medians (subtracting sample median). Fold-change differences in the quantity of proteins detected in different samples were calculated by fitting a linear regression model that takes into account donor variability at both the peptide and protein levels (13, 14).

Reactome graphs and pathway analysis. Pathway representation analysis of protein-level fold-changes was carried out using the Reactome Pathway Analysis tool (Pathway browser version 3.6, Reactome database release 74) (15, 16). Significantly represented pathways at the FDR-corrected 5% level were coloured according to the mean $\log_2$-fold change of proteins within the pathway. Statistical enrichment analysis was carried out using the PANTHER gene list analysis tool (17) to identify over- or under-enriched pathways with an FDR-corrected p-value ≤ 0.05.

Modularity analysis. The 332 protein human chaperome (18) was modelled as a weighted undirected network in order to discern its community structure. Chaperones with at least one interaction satisfying the highest confidence filter from STRING database (version 11) (19) were used as the nodes of the network, whilst these highest confidence interactions between chaperones were used as undirected edges in the network. Edges between nodes were weighted according to the STRING interaction score between the respective chaperones. The network modularity, $Q^W \epsilon \, [-0.5,1]$, was used to give a measure of how well the network separated into non-overlapping communities of highly-interconnected nodes. A network with edges distributed at random would score a modularity of zero, indicating no presence of community structure, whilst higher scores would indicate more intramodular edges than would be expected at random. The maximal modularity score was calculated using methods described previously (20, 21):

$$Q^w = \frac{1}{l^W} \sum_{i,j \in N} \left[w_{ij} - \frac{k_i^W k_j^W}{l^W} \right] \delta_{m_i,m_j} \qquad \text{(EQN 1)}$$

Where i and j denote two elements of the set of all nodes N, connected by an edge of weight $w_{ij} \epsilon (0,1]$. $l^W = \sum_{i,j \epsilon N} w_{ij}$ is the sum of all weights in the network; $k_i^W = \sum_{j \epsilon N} w_{ij}$ is the weighted degree of node i; and $\delta_{m_i,m_j} = \begin{cases} 0 & i \neq j \\ 1 & i = j \end{cases}$ is the Kronecker delta function, where m_i is the module containing node i. 942 interactions were identified between 181 chaperone proteins. These interactions were used to generate an adjacency matrix A, whose elements A_{ij} were the weights of edges between two elements $i, j \in N$. To calculate the modularity of the network, A was used as the input for the MATLAB Brain Connectivity Toolbox (21). The maximal modularity of the human chaperome was found to be $Q^w = 0.5328$, with a community structure consisting of 19 modules. The 5 modules with the most proteins seen in our label-free mass spectrometry dataset were investigated and named according to the function of chaperones within the modules.

Linear modelling. Linear regression models were fit to data in MATLAB using the *fitlm* function (R2015a, MathWorks) using the equation:

$$y_{dtp} = \beta_0 + \beta_d x_d + \beta_t x_t + \beta_p x_p + \varepsilon_{dtp} \qquad \text{(EQN 2)}$$

The response variable y_{dtp} (e.g. HSPA1A concentration) was modelled as being dependent on donor *d*, temperature *t* (categorical variable, either with or without heat shock), and passage number *p* (categorical variable, either early passage (EP) or late passage (LP, i.e. senescent), plus an error term, ε_{dtp}.

Rate equations used to describe the heat stress response. We established a model of the heat stress response that considered the concentrations of six key species: HSF1 (active HSF1 protein), HSPA1A, CHIP (E3 ubiquitin-protein ligase CHIP protein), MFP (misfolded protein), HSPA1A-HSF1 (HSPA1A in complex with inactive HSF1), and HSPA1A-MFP (HSPA1A bound to a misfolded protein). The model was based on the following seven reactions, with reaction rates k_i:

HSPA1A synthesis, $[HSF1] \xrightarrow{k_1} [HSF1] + [HSPA1A]$

HSF1 inactivation through HSPA1A binding, $[HSF1] + [HSPA1A] \xrightarrow{k_2} [HSF1 \cdot HSPA1A]$

HSPA1A binding to a misfolded protein, $[HSPA1A] + [MFP] \xrightarrow{k_3} [HSPA1A \cdot MFP]$

$[HSPA1A \cdot HSF1] + [MFP] \xrightarrow{k_3} [HSF1] + [HSPA1A \cdot MFP]$

CHIP-induced HSPA1A turnover, $[HSPA1A] + [CHIP] \xrightarrow{k_4} [CHIP]$

$[HSPA1A \cdot HSF1] + [CHIP] \xrightarrow{k_4} [CHIP] + [HSF1]$

HSPA1A-mediated protein refolding, $[HSPA1A \cdot MFP] \xrightarrow{k_5} [HSPA1A]$

CHIP-induced turnover of misfolded protein bound to HSP7A1A, $[HSPA1A \cdot MFP] + [CHIP] \overset{k_6}{\rightarrow} [HSPA1A] + [CHIP]$

Protein misfolding, $\emptyset \overset{k_7}{\rightarrow} [MFP]$

HSPA1A synthesis was modelled using a Hill function,

$$k_1 = k \frac{[HSF]^n}{K_d^3 + [HSF]^n} \qquad \text{(EQN 3)}$$

Where the Hill coefficient, $n = 3$, was chosen to reflect HSF1 trimerisation; $k = \lim_{[HSF1] \to \infty} k_1$ was the maximal transcription rate and K_d the HSF1 dissociation constant.

Initial conditions, constraints and running of the model. Initial conditions and constraints were based on experimental and literature-sourced results. Concentrations of component species were set relative to HSF1, quasi-arbitrarily set at 0.1 μM, based on previous estimates (22). It was assumed that there would be no substantial difference between the binding affinity of HSPA1A to HSF1 or client proteins, such that $k_2 \approx k_3$. The turnover time for a protein via the proteasome was assumed to be linearly proportional to its length in amino acids. To incorporate this, the ratio between the number of amino acids in HSPA1A and the median length of a human protein (23) was used to estimate the relative time taken for turnover following ubiquitination, $k_6 : k_4 = 641 : 375$. Parameters were optimised using the MATLAB *fminsearch* function to minimise the total sum of squared errors (SSE) between *in vitro* measurements of HSPA1A concentration *and in silico* values calculated at matched timepoints.

The temporal dynamics of the heat shock response were modelled using MATLAB (R2015a, MathWorks). All populations were recalculated at discrete time intervals using ordinary differential equations (ODE), except for $HSP70$ which was calculated using a delay differential equation (DDE). This accounted for the time delay ($\tau \sim U[60, 180]$ minutes) between transcription and translation (24). The ODE/DDEs were evaluated every (simulated) 0.01 minutes. A proteotoxic stress comparable with heat shock was simulated by multiplying the reaction rate k_7 by another optimised parameter, $\alpha > 1$ for 120 minutes.

Protein labeling with monobromobimane (mBBr). Media was removed from cells in T75 flasks (Corning) either pre-stress or immediately post-stress and cells were washed with PBS. Cells were then labelled by incubation with 5 mL of 400 µM monobromobimane (mBBr; Sigma-Aldrich) in PBS at 37 °C for 10 min. Following labelling, 5 mL of 2 mM glutathione in PBS was added to quench the reaction. The quenched mBBr solution was removed and cells washed with PBS, before harvesting with trypsin for MS analysis, as described above. When searching MS data, the peptide database was modified to search for mBBr-adducts to cysteine (monoisotopic mass changes, 133.053 and 150.056 Da). mBBr labelled peptides were filtered to only include sequences from reviewed protein annotations (25) and labelled peptides with missed cleavages were summed with their fully tryptic counterparts. 359 unique mBBr labelled peptides were detected. The $\log_2$ fold-change in labelling across samples was normalised to the respective $\log_2$ fold-change in protein abundance. A Wilcoxon signed-rank test was used to determine whether the mBBr-labelling profile changed between samples at the 95% confidence level.

RNA-sequencing. All reagents and solutions were supplied with the TruSeq Stranded mRNA assay (Illumina, Inc.,#20020594). Quality and integrity of total RNA samples from five biological replicates were assessed using a TapeStation 2200 (Agilent Technologies). To generate libraries, total RNA (0.1 – 4.0 µg) was used as input material from which polyadenylated mRNA was purified using poly-T, oligo-attached, magnetic beads. The mRNA was then fragmented using divalent cations under elevated temperature and then reverse transcribed into first strand cDNA using random primers. Second strand cDNA was then synthesised using DNA Polymerase I and RNase H. Following a single 'A' base addition, adapters were ligated to the cDNA fragments, and the products then purified and enriched by PCR to create the final cDNA library. Adapter indices were used to multiplex libraries, which were pooled prior to cluster generation using a cBot instrument (Illumina, Inc.). The loaded flow-cell was then paired-end sequenced (76 + 76 cycles, plus indices) on a HiSeq4000 instrument (Illumina, Inc.). Finally, the output data was demultiplexed (allowing one mismatch) and BCL-to-Fastq conversion was performed using bcl2fastq software (Illumina Inc., version 2.20.0.422).

Analysis of sequencing data. The quality of RNA-sequencing data was assessed using Phred quality scores, $Q = -10 \log_{10} P$, where P is the base calling error probability (26). Unmapped paired-end sequences were assessed by FastQC software (Babraham Bioinformatics). Sequence adapters were removed and reads were quality trimmed (to the threshold $Q \geq 20$) using Trimmomatic (version 0.36) (27). The reads were mapped against the reference human genome (hg38) and counts per gene were calculated using annotation from GENCODE 36 using STAR (version 2,7,2b) (28). Normalisation, principal component analysis, and differential expression was calculated in DESeq2 (version 1.20.0) using default settings (29). The experiment was treated as a standard 2 x 2 design and data was fitted to a linear regression model. Where figures containing -omic data are windowed for ease of interpretation, all plots contain ≥ 99% of data points.

SUPPLEMENTAL FIGURES

Figure S1.

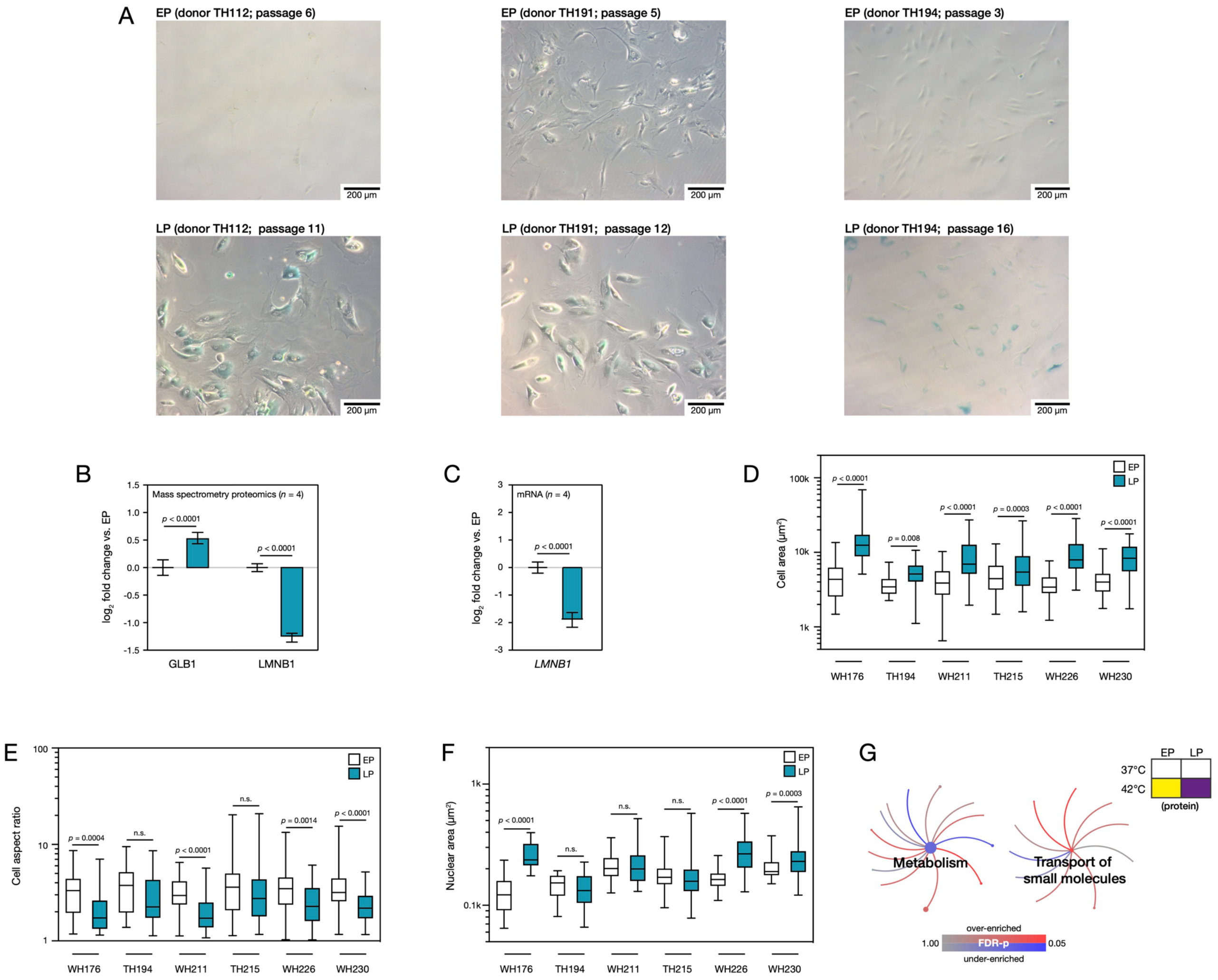

Figure S1. Characteristics of senescence in primary human mesenchymal stem cells (hMSCs) cultured to late passage (LP) were increased relative to those in donor-matched cells at early passage (EP). LP cells were examined at the passage number where onset of senescence occurred i.e. further cell proliferation was halted. This point was found to vary between donors (see SI Materials and Methods). Comparisons were made between donor-matched samples of senescent LP and EP cells: (**A**) LP hMSCs showed positive staining for β-galactosidase; representative images from n = 3 primary donors. (**B**) Quantitative mass spectrometry proteomics (Fig. 1A) showed a corresponding significant increase in β-galactosidase protein (GLB1, $p < 0.0001$) in LP vs. EP cells, and significant loss of the senescence marker lamin-B1 (LMNB1, $p < 0.0001$) (30). Log_2 fold-changes and significance values corrected for Benjamini-Hochberg false discovery rate (BH-FDR) were calculated as described previously (14); n = 4 primary donors. (**C**) RT-qPCR also showed a corresponding significant decrease of *LMNB1* transcript in LP vs. EP hMSCs ($p < 0.0001$ established by t-test; normalisation to housekeeping gene *PPIA*; n = 4 primary donors). (**D**) An analysis of cell morphology showed that LP hMSCs had significantly larger spread areas than EP cells, (p-values from unpaired t-tests in LP vs. EP). (**E**) LP hMSCs generally exhibited lower aspect ratios than EP cells, (p-values from Mann-Whitney tests in LP vs. EP). Increased cell spreading and loss of spindle-like morphology are characteristic of senescence (31, 32). (**F**) Nuclear area did not show a robust trend in LP vs. EP hMSCs (p-values from unpaired t-tests in LP vs. EP). In panels (D)-(F), box-whisker plots show means, quartiles and data spread; minimum 16 cells imaged per condition. Representative images of EP and LP hMSCs with cell morphology visualised by DAPI and phalloidin staining can be found in Figs. 3A, 4D and Supplemental Fig. S3H (-2 hour time-points, i.e. pre-stress). (**G**) Expanded view of “metabolism” and “transport of small molecule” pathways, following Reactome pathway analysis of the proteins shown in Figure 1D of the main paper, comparing the responses of EP and LP hMSCs subjected to heat shock (16). Significantly represented pathways (FDR-corrected p-value < 0.05) are shown in colours corresponding to the mean log_2 fold-change of proteins in the pathway. The sub-pathways “metabolism of carbohydrates” and “iron uptake and transport” were significantly over-enriched in LP hMSCs, but there were no overall significant changes to the parent pathways.

Figure S2

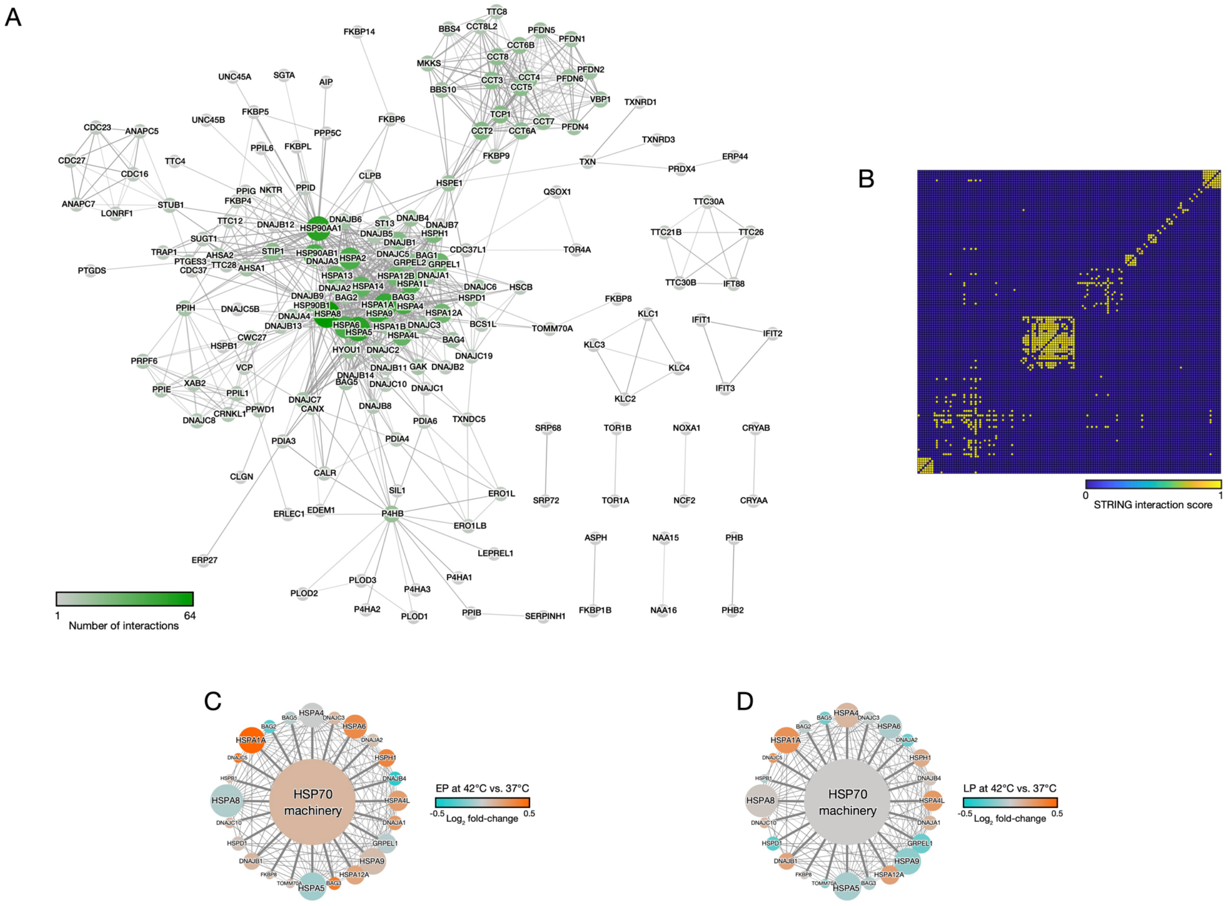

Figure S2. Application of network analysis to define functional groups within the human chaperome. (**A**) The 332 protein human chaperone network (33), with nodes coloured according to chaperone degree as a measure of importance to the function of the network (21). The most highly connected proteins in the network were: HSPA8 (*degree = 64*); HSP90AA1 (*53*); HSPA5 (*52*); HSPA9 (*48*); and, HSPA1A (*47*). (**B**) The adjacency matrix of the human chaperone network following modularity analysis. Rows and columns are the 181 nodes in the network, with entries representing the STRING interaction score (19). Interactions have been filtered to only include those of the highest confidence (score ≥ 0.9). (**C**) Network showing changes to individual proteins in the HSP70 module of Fig. 2E in the main paper. (**D**) Changes to individual proteins in the HSP70 module of Fig. 2F in the main paper. In panels (C) and (D), node size is indicative of chaperone degree, while edge weight indicates interaction score between chaperones.

Figure S3.

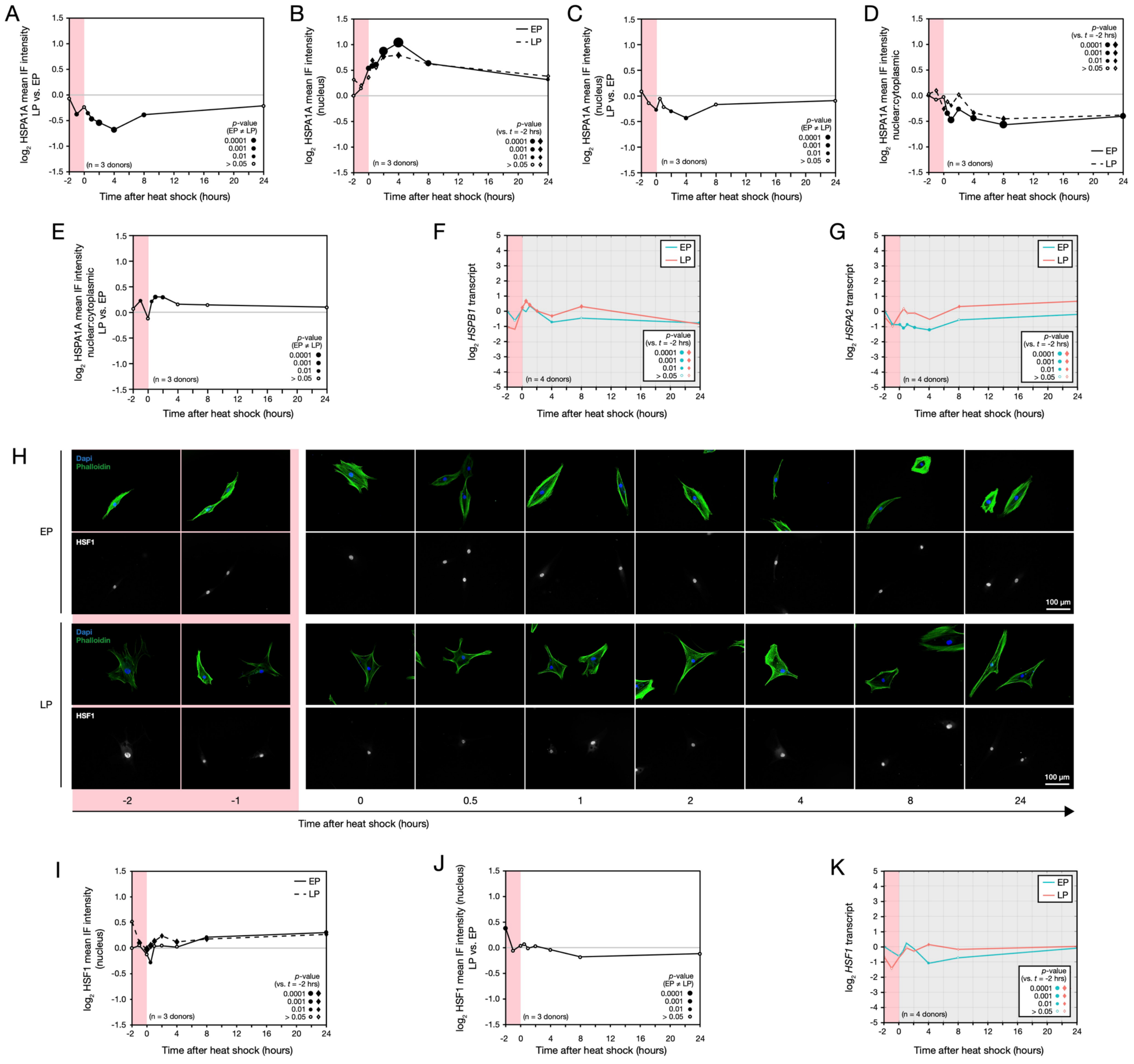
A
B
C
D
E
F
G
H
I
J
K
log2 HSPA1A mean IF intensity LP vs. EP
log2 HSPA1A mean IF intensity (nucleus)
log2 HSPA1A mean IF intensity (nucleus) LP vs. EP
log2 HSPA1A mean IF intensity nuclear:cytoplasmic
log2 HSPA1A mean IF intensity nuclear:cytoplasmic LP vs. EP
log2 HSPB1 transcript
log2 HSPA2 transcript
log2 HSF1 mean IF intensity (nucleus)
log2 HSF1 mean IF intensity (nucleus) LP vs. EP
log2 HSF1 transcript
Time after heat shock (hours)
(n = 3 donors)
(n = 4 donors)
p-value (EP ≠ LP)
p-value (vs. t = -2 hrs)
0.0001
0.001
0.01
> 0.05
EP
LP
Dapi
Phalloidin
HSF1
100 µm
-2
-1
0
0.5
1
2
4
8
24

Figure S3. Analysis of heat shock protein 70 kDa (HSPA1A), heat shock factor 1 (HSF1) and chaperone transcripts, in early passage (EP) and late passage (LP) human mesenchymal stem cells (hMSCs) subjected to heat treatment. hMSCs were imaged before, during and over a 24-hour period following a 2-hour heat treatment at 42 °C (see Fig. 3A in the main paper). Mean HSPA1A intensities were determined in nuclear and cytoplasmic cellular regions, defined by areas of DAPI and phalloidin staining, respectively. (**A**) Ratios of mean intensities of HSPA1A in LP vs. EP hMSCs before, during and after heat shock. (**B**) Levels of HSPA1A protein in the nuclei of EP and LP hMSCs before, during and after heat stress. (**C**) Ratios of mean intensities of HSPA1A in the nuclei of LP vs. EP hMSCs subjected to heat stress. (**D**) Nuclear-to-cytoplasmic ratios of HSPA1A in EP and LP hMSCs subjected to heat stress. (**E**) Nuclear-to-cytoplasmic ratios of HSPA1A in LP vs. EP hMSCs subjected to heat stress. (**F**) Levels of heat shock protein beta-1 transcript (*HSPB1*) in EP and LP hMSCs before, during and following heat stress. (**G**) Levels of heat shock-related 70 kDa protein 2 transcript (*HSPA2*) in EP and LP hMSCs before, during and following heat stress. (**H**) Representative immunofluorescence (IF) images of HSF1 in EP and LP hMSCs before, during and for 24 hours following a 2-hour heat treatment at 42 °C. (**I**) Quantification of levels of HSF1 protein in the nuclei of EP and LP hMSCs before, during and after heat stress. (**J**) Ratios of mean intensities of HSF1 in the nuclei of LP vs. EP hMSCs subjected to heat stress. (**K**) Levels of heat shock factor 1 transcript (*HSF1*) in EP and LP hMSCs before, during and following heat stress. In panels (A), (C), (E) and (J), point size indicates significance where EP $\neq$ LP, with solid points showing $p < 0.05$ (ANOVA, $n = 3$ primary donors). In figure panels (B), (D) and (I), point size indicates the significance of changes vs. pre-heating, with solid points showing $p < 0.05$ (ANOVA, $n = 3$ primary donors). In panels (F), (G) and (K), point size indicates significance of change in transcript level vs. pre-heat shock, with solid points showing $p < 0.05$ (ANOVA, $n = 4$ primary donors).

Figure S4.

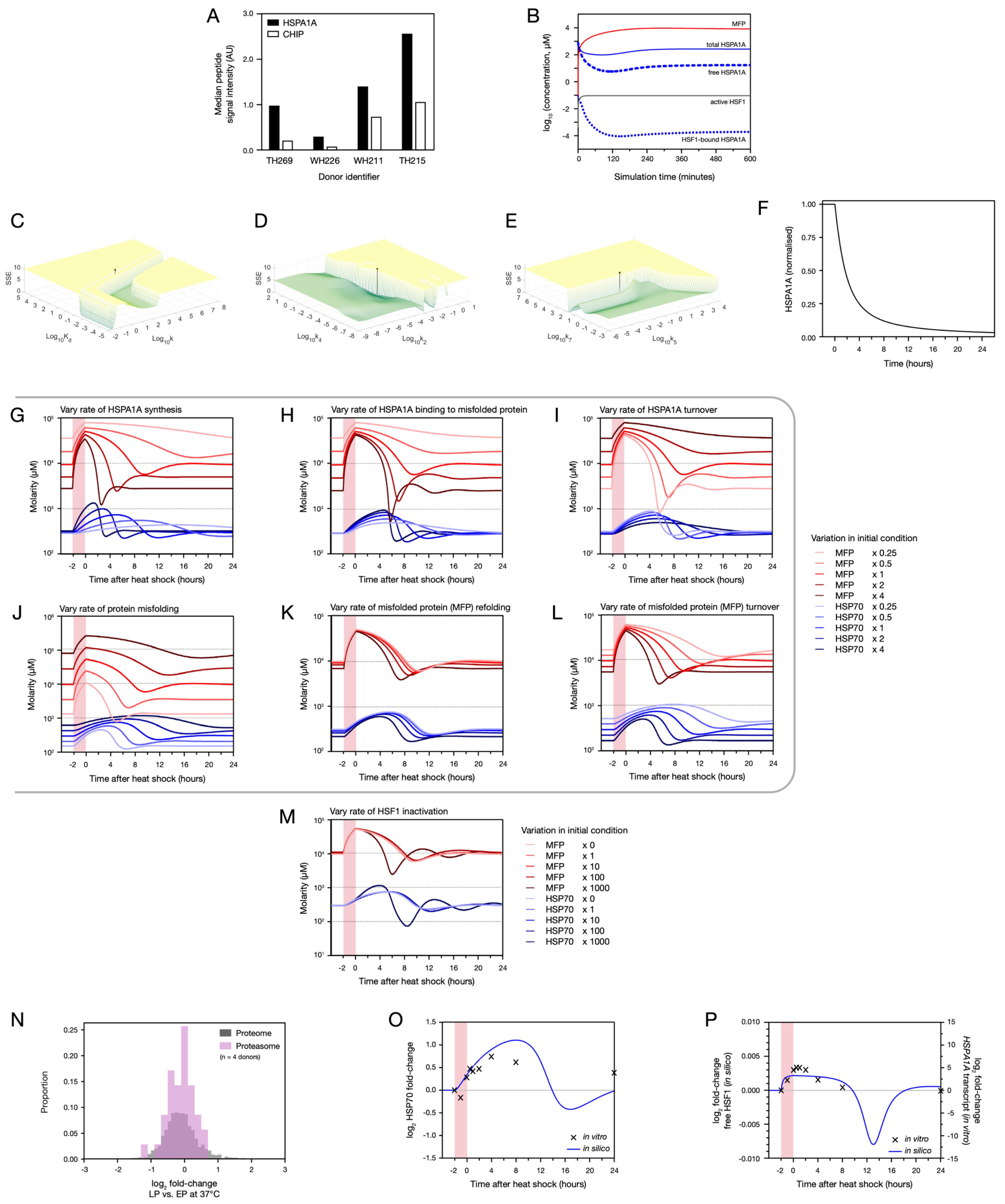
A
HSPA1A
CHIP
Median peptide signal intensity (AU)
TH269
WH226
WH211
TH215
Donor identifier
B
MFP
total HSPA1A
free HSPA1A
active HSF1
HSF1-bound HSPA1A
Simulation time (minutes)
C
D
E
SSE
F
HSPA1A (normalised)
Time (hours)
G
Vary rate of HSPA1A synthesis
H
Vary rate of HSPA1A binding to misfolded protein
I
Vary rate of HSPA1A turnover
J
Vary rate of protein misfolding
K
Vary rate of misfolded protein (MFP) refolding
L
Vary rate of misfolded protein (MFP) turnover
Molarity (µM)
Time after heat shock (hours)
Variation in initial condition
MFP x 0.25
MFP x 0.5
MFP x 1
MFP x 2
MFP x 4
HSP70 x 0.25
HSP70 x 0.5
HSP70 x 1
HSP70 x 2
HSP70 x 4
M
Vary rate of HSF1 inactivation
Variation in initial condition
MFP x 0
MFP x 1
MFP x 10
MFP x 100
MFP x 1000
HSP70 x 0
HSP70 x 1
HSP70 x 10
HSP70 x 100
HSP70 x 1000
N
Proteome
Proteasome
(n = 4 donors)
Proportion
log2 fold-change
LP vs. EP at 37°C
O
log2 HSP70 fold-change
in vitro
in silico
P
log2 fold-change free HSF1 (in silico)
log2 fold-change HSPA1A transcript (in vitro)
in vitro
in silico

Figure S4. Results supporting development and characterisation of an ordinary / delayed differential equation (ODE/DDE) model of the cellular response to heat stress. (**A**) HSPA1A and CHIP protein signal intensities quantified by mass spectrometry, in the absence of stress, in early passage (EP) human mesenchymal stem cells (hMSCs) from four donors. (**B**) Convergence of populations in the ODE/DDE model to a stable equilibrium prior to stress. (**C-E**) Demonstration of the optimisation of free model parameters. The ODE/DDE model was used to simulate the stress response over a range of parameter values, and the sum of squared errors (SSE) compared to experimentally derived data (measurements of HSPA1A in EP hMSCs by immunofluorescence) at each parameter value. The parameter values chosen by optimisation are shown overlaid in black over the SSE values. (**F**) Decay of HSPA1A levels simulated by removing HSPA1A synthesis from the ODE/DDE model at equilibrium. The *in silico* half-life of HSPA1A was 100 minutes. Changes to HSPA1A and misfolded protein (MFP) dynamics in the ODE/DDE model were analysed upon modification of the following parameters: (**G**) Rate of HSPA1A synthesis; (**H**) Rate of HSPA1A binding to misfolded protein; (**I**) Rate of HSPA1A turnover; (**J**) Rate of protein misfolding; (**K**) Rate of refolding of MFP; (**L**) Rate of MFP turnover; (**M**) Rate of HSF1 inactivation. In figures (G)-(M), the legend indicates changes to concentrations of HSPA1A (in red) and MFP (in blue) upon fold change to the indicated parameter (i.e., '0.5x', halved; '2x', doubled), relative to rates in SI Table S7. (**N**) Levels of proteins identified as constitutive of the proteasome, compared to the whole proteome in LP vs. EP hMSCs in the absence of heat shock, determined from mass spectrometry analysis (Figure 1 in the main paper). Although 20% of proteasomal components were significantly changed between EP and LP (FDR-corrected $p < 0.05$), there was no overall significant change in levels of proteasomal components vs. the proteome (FDR-corrected $p = 0.99$). (**O**) ODE/DDE model simulation of the stress response with adjustments made to resemble senescent cells. The *in silico* model was given sufficient time to reach a stable equilibrium, before a proteotoxic stress was simulated by increasing the rate at which misfolded proteins were generated within the model for 120 minutes. At each time interval, the *in silico* concentration of HSPA1A was recorded, and is shown overlaid with data acquired by immunofluorescence from late passage (LP) hMSCs *in vitro* (see Fig. 3B of the main paper). (**P**) As (O), but showing concentrations of modelled active HSF1 overlaid, with a scaling factor, onto experimentally-derived *HSPA1A* transcript levels in EP hMSCs (see Fig. 3C of the main paper).

Figure S5.

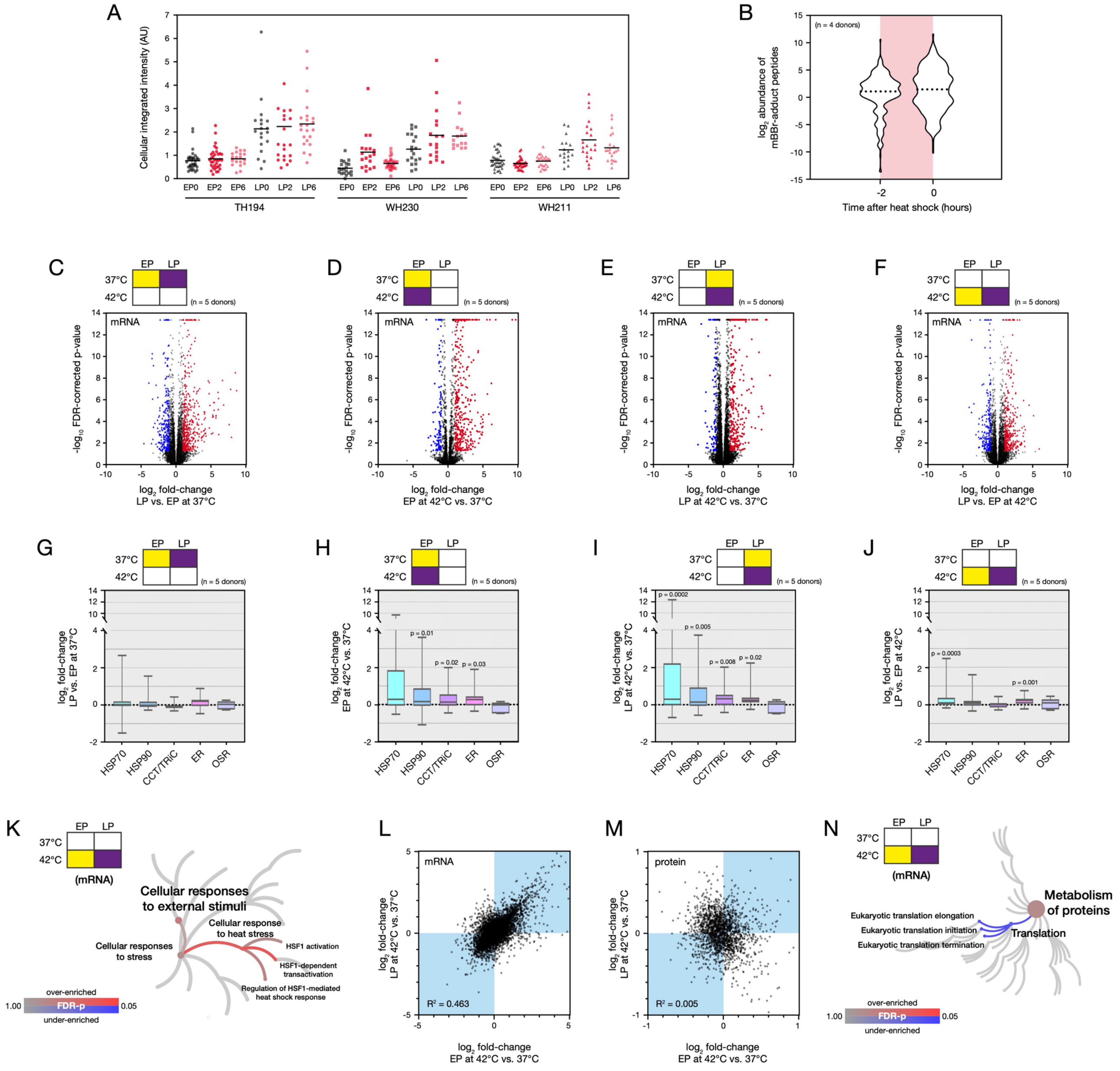
A
Cellular integrated intensity (AU)
EP0 EP2 EP6 LP0 LP2 LP6
TH194
WH230
WH211
B
(n = 4 donors)
log2 abundance of mBBr-adduct peptides
Time after heat shock (hours)
C
D
E
F
EP LP
37°C
42°C
(n = 5 donors)
mRNA
-log10 FDR-corrected p-value
log2 fold-change
LP vs. EP at 37°C
EP at 42°C vs. 37°C
LP at 42°C vs. 37°C
LP vs. EP at 42°C
G
H
I
J
log2 fold-change LP vs. EP at 37°C
log2 fold-change EP at 42°C vs. 37°C
log2 fold-change LP at 42°C vs. 37°C
log2 fold-change LP vs. EP at 42°C
HSP70
HSP90
CCT/TRiC
ER
OSR
p = 0.01
p = 0.02
p = 0.03
p = 0.0002
p = 0.005
p = 0.008
p = 0.0003
p = 0.001
K
(mRNA)
Cellular responses to external stimuli
Cellular response to heat stress
Cellular responses to stress
HSF1 activation
HSF1-dependent transactivation
Regulation of HSF1-mediated heat shock response
over-enriched
FDR-p
under-enriched
1.00
0.05
L
mRNA
R² = 0.463
log2 fold-change LP at 42°C vs. 37°C
log2 fold-change EP at 42°C vs. 37°C
M
protein
R² = 0.005
N
Metabolism of proteins
Translation
Eukaryotic translation elongation
Eukaryotic translation initiation
Eukaryotic translation termination

Figure S5. Effects of a senescence-supressed response to heat stress on protein aggregation and transcriptional regulation in human mesenchymal stem cells (hMSCs). (**A**) Quantification of protein aggregates by ProteoStat staining in early passage (EP) and late passage (LP) hMSCs from three donors, pre-stress (black points, t = 0 hrs), immediately following a 2-hour heat shock treatment at 42 °C (red points, t = 2 hrs), and 4 hours after the heat treatment (pink points, t = 6 hrs). (**B**) Abundances of monobromobimane (mBBr)-tagged peptides in mass spectrometry data pre-stress and immediately following a 2-hour heat shock treatment at 42 °C. Dotted lines indicate sample medians. RNA-sequencing was used to compare the transcriptomes of donor-matched early passage (EP) and senescent late passage (LP) hMSCs with and without a 2-hour heat shock treatment at 42 °C. Volcano plots showing the distribution of changes in the abundance of (**C**) 15782 transcripts in LP vs. EP hMSCs in the absence of heat shock; (**D**) 17243 transcripts in EP hMSCs with and without heat shock; (**E**) 16550 transcripts in LP hMSCs with and without heat shock; (**F**) 15378 transcripts in LP vs. EP hMSCs, both subjected to heat shock. In figure panels (C)-(F), red and blue points satisfy a p-value < 0.05 and an absolute $\log_2$ fold-change > 1; n = 5 primary donors. Changes to transcript levels of members of chaperome modules (defined in Fig. 2 of the main paper) (**G**) in LP vs. EP hMSCs in the absence of heat shock; (**H**) in EP hMSCs with and without heat shock; (**I**) in LP hMSCs with and without heat shock; (**J**) in LP vs. EP hMSCs subjected to heat shock. In figure panels (G)-(J), box-whisker plots show medians, quartiles and range; p-values from ANOVA; n = 5 primary donors. (**K**) Transcript-level changes to the "Cellular responses to external stimuli" pathway family between stressed EP and LP populations. Product-moment correlation coefficients were calculated to compare the similarity between the EP and LP stress responses for (**L**) transcriptomic and (**M**) proteomic data. (**N**) Transcript-level changes to the "Metabolism of proteins" pathway family between stressed EP and LP populations.

Figure S6.

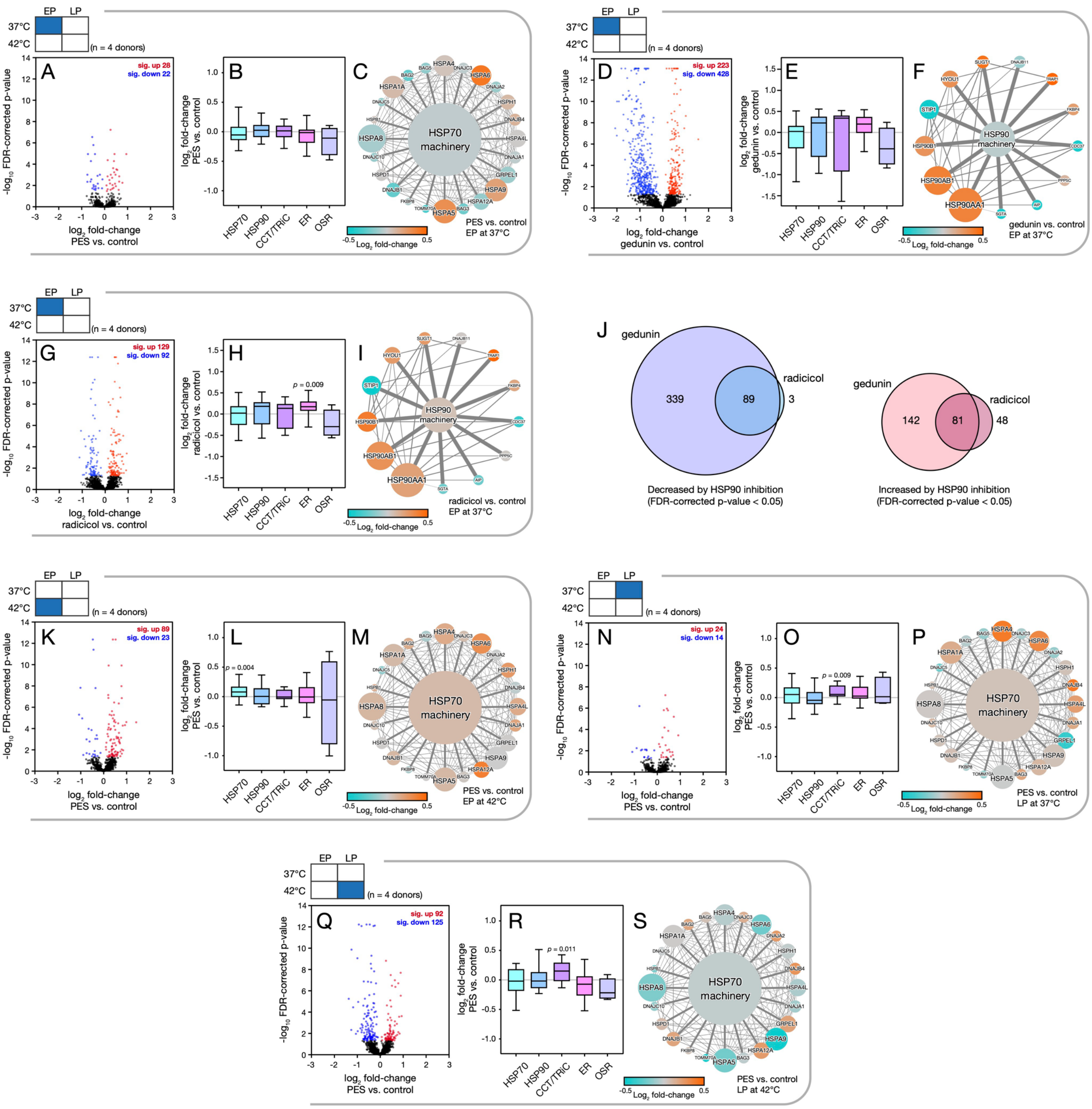
A
sig. up 28
sig. down 22
log2 fold-change PES vs. control
-log10 FDR-corrected p-value
B
C
HSP70 machinery
PES vs. control EP at 37°C
D
sig. up 223
sig. down 428
log2 fold-change gedunin vs. control
E
F
HSP90 machinery
gedunin vs. control EP at 37°C
G
sig. up 129
sig. down 92
log2 fold-change radicicol vs. control
H
p = 0.009
I
radicicol vs. control EP at 37°C
J
gedunin
radicicol
339
89
3
Decreased by HSP90 inhibition (FDR-corrected p-value < 0.05)
142
81
48
Increased by HSP90 inhibition (FDR-corrected p-value < 0.05)
K
sig. up 89
sig. down 23
L
p = 0.004
M
PES vs. control EP at 42°C
N
sig. up 24
sig. down 14
O
p = 0.009
P
PES vs. control LP at 37°C
Q
sig. up 92
sig. down 125
R
p = 0.011
S
PES vs. control LP at 42°C
(n = 4 donors)
EP
LP
37°C
42°C
HSP70
HSP90
CCT/TRiC
ER
OSR
Log2 fold-change

Figure S6. Proteomic analysis of primary human mesenchymal stem cells (hMSCs) treated with chaperone inhibitors. (**A**) Volcano plot showing the effect of HSP70-inhibitor 2-phenylethynesulfonamide (PES) on early passage (EP) hMSCs at 37 °C. (**B**) PES treatment caused no significant changes to chaperome modules in EP hMSCs. (**C**) Network showing changes to individual proteins in the HSP70 module of figure panel (B). (**D**) Volcano plot showing the effect of HSP90-inhibitor gedunin on EP hMSCs at 37 °C. (**E**) Gedunin treatment caused no significant changes to chaperome modules in EP hMSCs. (**F**) Network showing changes to individual proteins in the HSP90 module of figure panel (E). (**G**) Volcano plot showing the effect of HSP90-inhibitor radicicol on EP hMSCs at 37 °C. (**H**) EP hMSCs treated with radicicol exhibited significant increase of the ER module (p = 0.009). (**I**) Network showing changes to individual proteins in the HSP90 module of figure panel (H). (**J**) Venn diagrams showing numbers of proteins significantly decreased or increased (FDR-corrected p-value < 0.05) by treatments with HSP90-inhibitors gedunin and radicicol. Gedunin was found to perturb the levels of a greater number of proteins – potentially due to increased potency or a greater number of off-target effects – but both treatments affected a common set of proteins. (**K**) Effect of PES on EP hMSCs subjected to a 2-hour heat shock at 42 °C. (**L**) EP hMSCs treated with PES in combination with heat shock exhibited significant increase of the HSP70 module (p = 0.004). (**M**) HSP70 network corresponding to panel (L). (**N**), (**O**) and (**P**) show conditions and analysis as in panels (A), (B) and (C), but applied to LP hMSCs. (**Q**), (**R**) and (**S**) show conditions and analysis as in panels (K), (L) and (M), but applied to LP hMSCs. In volcano plots, red and blue points satisfy a p-value < 0.05. p-values were calculated using empirical Bayes-modified t-tests with Benjamini–Hochberg false discovery rate correction (14); n = 4 primary donors. Significance of changes to modules was determined from ANOVA testing. In network diagrams, node size is indicative of chaperone degree, while edge weight indicates interaction score between chaperones. The central node is coloured according to the mean abundance change of chaperones associated with the module. Descriptions of the chaperone modules can be found in Fig. 2A of the main paper.

SUPPLEMENTAL TABLE

Parameter	Description	Value	Unit
k	Maximal HSPA1A transcription rate	6.4294×10^{2}	min^{-1}
k	(Modified value in senescent cells)	4.5865×10^{2}	min^{-1}
n	Hill coefficient	3	(dimensionless)
K_d	HSF1 dissociation constant	3.0534	μM
k_2	HSF1-HSPA1A binding rate	3.9827×10^{-4}	$\mu M^{-1} min^{-1}$
k_3	HSPA1A-MFP binding rate	3.9827×10^{-4}	$\mu M^{-1} min^{-1}$
k_4	CHIP-mediated HSPA1A turnover rate	1.2400×10^{-3}	$\mu M^{-1} min^{-1}$
k_5	HSPA1A-mediated MFP refolding rate	5.0199×10^{-2}	min^{-1}
k_6	CHIP-mediated MFP turnover rate	2.1200×10^{-3}	$\mu M^{-1} min^{-1}$
k_7	Rate of protein misfolding at 37 °C	69.1068	$\mu M\ min^{-1}$
λ	Rate of protein misfolding at 42 °C	4.2651×10^{2}	$\mu M\ min^{-1}$
t	Simulation time elapsed		min
τ	HSF1 activation/HSPA1A synthesis delay	$\sim U[60, 180]$	min

Supplemental Table S7. Optimised and approximated parameters for the ordinary/delay differential equation (ODE/DDE) mathematical model of the heat stress response. MFP = misfolded protein; CHIP = E3 ubiquitin-protein ligase CHIP.

SUPPLEMENTAL REFERENCES

1. J. I. J. Leu, J. Pimkina, A. Frank, M. E. Murphy, D. L. George, A small molecule inhibitor of inducible heat shock protein 70. *Mol. Cell* **36**, 15-27 (2009).

2. J. I. J. Leu, J. Pimkina, P. Pandey, M. E. Murphy, D. L. George, HSP70 inhibition by the small-molecule 2-phenylethynesulfonamide impairs protein clearance pathways in tumor cells. *Mol. Cancer Res.* **9**, 936-947 (2011).

3. J. I. J. Leu *et al.*, Inhibition of stress-inducible HSP70 impairs mitochondrial proteostasis and function. *Oncotarget* **8**, 45656-45669 (2017).

4. J. Yang, W. B. Gong, S. Wu, H. Zhang, S. Perrett, PES inhibits human-inducible Hsp70 by covalent targeting of cysteine residues in the substrate-binding domain. *J. Biol. Chem.* **296** (2021).

5. C. A. Patwardhan *et al.*, Gedunin inactivates the co-chaperone p23 protein causing cancer cell death by apoptosis. *J. Biol. Chem.* **288**, 7313-7325 (2013).

6. S. M. Roe *et al.*, Structural basis for inhibition of the Hsp90 molecular chaperone by the antitumor antibiotics radicicol and geldanamycin. *J. Med. Chem.* **42**, 260-266 (1999).

7. S. Strassburg, S. M. Richardson, A. J. Freemont, J. A. Hoyland, Co-culture induces mesenchymal stem cell differentiation and modulation of the degenerate human nucleus pulposus cell phenotype. *Regen. Med.* **5**, 701-711 (2010).

8. R. L. Matts *et al.*, A systematic protocol for the characterization of Hsp90 modulators. *Bioorg. Med. Chem.* **19**, 684-692 (2011).

9. T. W. Schulte *et al.*, Interaction of radicicol with members of the heat shock protein 90 family of molecular chaperones. *Mol. Endocrinol.* **13**, 1435-1448 (1999).

10. L. Kamentsky *et al.*, Improved structure, function and compatibility for CellProfiler: modular high-throughput image analysis software. *Bioinformatics* **27**, 1179-1180 (2011).

11. K. J. Livak, T. D. Schmittgen, Analysis of relative gene expression data using real-time quantitative PCR and the 2(T)(-Delta Delta C) method. *Methods* **25**, 402-408 (2001).

12. Y. Benjamini, Y. Hochberg, Controlling the false discovery rate - a practical and powerful approach to multiple testing. *J. R. Stat. Soc. Ser. B-Stat. Methodol.* **57**, 289-300 (1995).

13. H. T. J. Gilbert *et al.*, Nuclear decoupling is part of a rapid protein-level cellular response to high-intensity mechanical loading. *Nat. Commun.* **10**, 4149-4115 (2019).

14. V. Mallikarjun, S. M. Richardson, J. Swift, BayesENproteomics: Bayesian elastic nets for quantification of peptidoforms in complex samples. *J. Proteome Res.* **19**, 2167-2184 (2020).

15. A. Fabregat *et al.*, The Reactome pathway knowledgebase. *Nucleic Acids Res.* **46**, D649-D655 (2018).

16. B. Jassal *et al.*, The Reactome pathway knowledgebase. *Nucleic Acids Res.* **48**, D498-D503 (2020).

17. H. Y. Mi *et al.*, PANTHER version 16: a revised family classification, tree-based classification tool, enhancer regions and extensive API. *Nucleic Acids Res.* **49**, D394-D403 (2021).

18. M. Brehme *et al.*, A chaperome subnetwork safeguards proteostasis in aging and neurodegenerative disease. *Cell Rep.* **9**, 1135-1150 (2014).

19. D. Szklarczyk *et al.*, STRING v11: protein-protein association networks with increased coverage, supporting functional discovery in genome-wide experimental datasets. *Nucleic Acids Res.* **47**, D607-D613 (2019).

20. M. E. J. Newman, Modularity and community structure in networks. *Proc. Natl. Acad. Sci. U. S. A.* **103**, 8577-8582 (2006).

21. M. Rubinov, O. Sporns, Complex network measures of brain connectivity: Uses and interpretations. *Neuroimage* **52**, 1059-1069 (2010).

22. R. Milo, What is the total number of protein molecules per cell volume? A call to rethink some published values. *Bioessays* **35**, 1050-1055 (2013).

23. L. Brocchieri, S. Karlin, Protein length in eukaryotic and prokaryotic proteomes. *Nucleic Acids Res.* **33**, 3390-3400 (2005).

24. A. F. Jarnuczak, M. G. Albornoz, C. E. Eyers, C. M. Grant, S. J. Hubbard, A quantitative and temporal map of proteostasis during heat shock in Saccharomyces cerevisiae. *Mol. Omics* **14**, 37-52 (2018).

25. A. Bateman *et al.*, UniProt: a worldwide hub of protein knowledge. *Nucleic Acids Res.* **47**, D506-D515 (2019).

26. B. Ewing, P. Green, Base-calling of automated sequencer traces using phred. II. Error probabilities. *Genome Res.* **8**, 186-194 (1998).

27. A. M. Bolger, M. Lohse, B. Usadel, Trimmomatic: a flexible trimmer for Illumina sequence data. *Bioinformatics* **30**, 2114-2120 (2014).

28. A. Dobin *et al.*, STAR: ultrafast universal RNA-seq aligner. *Bioinformatics* **29**, 15-21 (2013).

29. M. I. Love, W. Huber, S. Anders, Moderated estimation of fold change and dispersion for RNA-seq data with DESeq2. *Genome Biol.* **15**, 38 (2014).

30. T. Shimi *et al.*, The role of nuclear lamin B1 in cell proliferation and senescence. *Genes Dev.* **25**, 2579-2593 (2011).

31. J. Liu, Y. Ding, Z. M. Liu, X. T. Liang, Senescence in mesenchymal stem cells: functional alterations, molecular mechanisms, and rejuvenation strategies. *Front Cell Dev Biol* **8** (2020).

32. A. Hernandez-Segura, J. Nehme, M. Demaria, Hallmarks of cellular senescence. *Trends Cell Biol.* **28**, 436-453 (2018).

33. M. Brehme *et al.*, A chaperome subnetwork safeguards proteostasis in aging and neurodegenerative disease. *Cell Rep.* **9**, 1135-1150 (2014).
